# Aging, Dauer, and Stature Phenotypes Are Conferred by Structure‐Directed Missense Mutations in the Endogenous AGE‐1/Phosphatidylinositol 3‐Kinase Catalytic Subunit

**DOI:** 10.1111/acel.70571

**Published:** 2026-06-18

**Authors:** You Wu, Tam Duong, Neal R. Rasmussen, Kent L. Rossman, David J. Reiner

**Affiliations:** ^1^ College of Medicine Texas A&M Health Science Center, Texas A&M University Houston Texas USA; ^2^ Institute of Biosciences and Technology Texas A&M Health Science Center, Texas A&M University Houston Texas USA; ^3^ Department of Surgery, School of Medicine University of North Carolina Chapel Hill North Carolina USA

**Keywords:** AGE‐1, aging, animal size, dauer diapause, LET‐60/Ras, lifespan, phosphatidylinositol 3‐kinase, vulval precursor cells

## Abstract

Phosphatidylinositol 3‐kinase (PI3K) integrates insulin/IGF signaling (IIS) and Ras inputs to control lifespan, metabolism and growth. Yet the organismal consequences of selective structural perturbations remain poorly understood. Using structure‐guided CRISPR/Cas9‐dependent genome editing, we dissected functions of AGE‐1, the sole Class IA PI3K catalytic subunit in *Caenorhabditis elegans* . An endogenously tagged AGE‐1, containing a long flexible linker, epitope and fluorescent tag, retained full activity, enabling visualization of native protein dynamics in vivo. A likely constitutively activating E630K substitution, modeled on oncogenic p110α alleles, markedly shortened lifespan and enhanced Ras‐dependent induction of primary vulval precursor cell (VPC) fate, confirming evolutionary conservation of PI3K activation mechanisms that directly modulate longevity and development. Structural modeling further guided mutation of AGE‐1 residues predicted to mediate Ras binding. Surprisingly, a putative AGE‐1 variant defective in Ras association, together with a complementary Ras effector‐binding mutation, produced enlarged animals with reduced dauer formation. These phenotypes reveal a previously unrecognized Ras>PI3K signaling axis that restrains somatic growth and promotes entry into diapause, counter to canonical IIS models. Together, these structure‐informed alleles show that discrete PI3K structural perturbations can differentially uncouple lifespan, growth, and developmental outcomes in vivo. By combining structural modeling with genome editing in a tractable aging model, this work establishes a framework for dissecting conserved signaling enzymes at single‐residue resolution and uncovers unexpected organismal roles for PI3K structure in coordinating growth and longevity.

## Graphical abstract

Structure‐guided missense mutations in the *C. elegans* AGE‐1/PI3Kinase catalytic subunit and its LET‐60/Ras‐binding interface reveal separable signaling outputs from the Ras‐bound PI3K complex. Enhanced canonical catalytic activity, conferred by a gain‐of‐function mutation modeled on oncogenic human p110⍺ PI3K variants, promotes aging, reproductive growth, and cell fate specification. In contrast, the Ras‐PI3K interaction selectively restrains animal growth and promotes dauer/diapause entry, demonstrating context‐dependent and functionally distinct outputs of PI3K signaling in vivo.

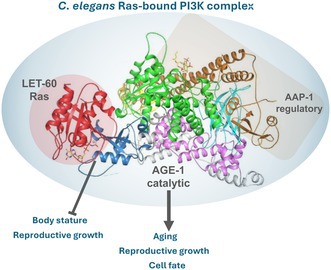

## 1 Introduction

Research across diverse systems—including human cohorts, rodent models, cell‐based assays, in vitro biochemistry/biophysics/reconstitution, computer modeling, and invertebrate organisms—can yield insights greater than the sum of their parts. Each platform has distinct strengths and limitations, and findings are often most powerful when integrated across disciplines. This perspective highlights the importance of what might be called “model discipline”: knowing where a model excels, where it falls short, and how technological advances may shift those boundaries.

Mammalian cell‐based systems permit rapid manipulations such as RNA interference (RNAi) or CRISPR/Cas9‐mediated depletion, often in high‐throughput formats. However, they are often limited by incomplete knockdown of endogenous proteins and gene redundancy, often with 2–4 paralogs per group in mammals (Dehal and Boore 2005). Mouse models can provide physiological relevance for human disease and lifespan but are costly and slow, complicating mechanistic linkage between protein structure and phenotype. Biochemical and biophysical studies precisely define molecular interactions but are typically divorced from organismal physiology.

Invertebrate models can bridge these gaps. *Caenorhabditis elegans* combines genetic tractability with the complex biological organization of a multicellular animal. “The Worm” is transparent and highly amenable to transgenesis and genome editing. With 959 somatic cells, 302 neurons, and a fully mapped cell lineage and neural connectome, *C. elegans* enables precise, in vivo analysis of conserved signaling networks and their impact on biology. These physical attributes are complemented by relatively little paralog redundancy while retaining broad conservation with disease‐relevant genes (Kim et al. 2018). The short generation time (~2.5 days), maximum lifespan (~18 days), and ease of culturing make *C. elegans* an ideal animal for exploration of mechanistic underpinnings of aging and lifespan while including a sophisticated genetic toolkit. Not surprisingly, discoveries in *C. elegans* have repeatedly advanced understanding of metabolism and aging (Kropp et al. 2021; Murphy and Hu 2013), beginning with the identification of *age‐1*, the first genetic determinant of lifespan in any system (Johnson 1990; Klass 1983).

Biochemical insights often drive genetic experiments in *C. elegans* . For example, catalytic residues defined in mammalian kinases and exchange factors for small GTPases can be tested in vivo to probe biological effects and functional conservation of proteins (Shin et al. 2019, 2018). When sequence conservation is limited, structural information becomes particularly valuable: three‐dimensional folds, domain interfaces, and higher‐order protein relationships are often preserved even when primary sequence identity diverges. Structural modeling thus enables rational design of targeted mutations for genome editing in model organisms.

We apply this approach to the catalytic subunit of Class IA phosphatidylinositol 3‐kinase (PI3K), encoded in mammals by PIK3CA, PIK3CB, and PIK3CD to produce p110α, p110β, and p110δ (hereafter PI3Kcat). PI3Kcat converts phosphatidylinositol (4,5)‐bisphosphate (PtdIns(4,5)P_2_, or PIP_2_) to phosphatidylinositol (3,4,5)‐trisphosphate (PtdIns(3,4,5)P_3_, or PIP_3_), which in turn activates downstream kinases PDK and AKT/PKB (Engelman et al. 2006; Fruman et al. 2017). In *C. elegans* , the sole PI3Kcat ortholog, AGE‐1, acts centrally within the conserved insulin/IGF‐like signaling (IIS) cascade, together with DAF‐2/InsR, AAP‐1 (regulatory PI3K subunit), IST‐1 (IRS1 adaptor), DAF‐18/PTEN, PDK‐1, and AKT‐1/2, to regulate lifespan, dauer diapause, and metabolic adaptation, processes central to human health (Murphy and Hu 2013; Partridge et al. 2011). Notably, these components were identified by classical forward genetic screens, rather than via structure‐guided hypotheses (Hu 2007). But the flourishing of CRISPR as an editing tool positions us to dissect functions of the AGE‐1 complex with a scalpel rather than a hammer.

PI3Kcat is activated by two inputs: direct recruitment to phosphotyrosines on the insulin/insulin‐like growth factor (IGF) receptor (InsR), a receptor tyrosine kinase (RTK) at the plasma membrane (PM), and by binding and recruitment to the PM by GTP‐bound Ras small GTPase (Rodriguez‐Viciana et al. 1994). Both mechanisms are conserved, and their balance influences downstream signaling and physiological outcomes. Structural studies in *Drosophila* and mouse PI3Kcat revealed residues required for Ras binding (Gupta et al. 2007; Orme et al. 2006), but their functional significance at the organismal level has not been tested in a system where lifespan and metabolic state can be directly assayed.

Here, we use structure‐guided CRISPR/Cas9‐dependent genome editing to dissect PI3Kcat function in vivo. We engineered into endogenous AGE‐1 protein (1) a C‐terminal fluorescent protein+epitope tag with a long, flexible linker to assess protein stability and enable live imaging without impairing function; (2) a gain‐of‐function substitution modeled on constitutively active p110α alleles from cancer, to test effects on lifespan and insulin/EGF growth factor‐related signaling; and (3) a mutation in the Ras‐binding domain (RBD), guided by structural modeling of Ras‐PI3Kcat interface. The RBD mutation increased body size and decreased dauer formation—phenotypes recapitulated by a complementary effector‐binding mutation in the effector‐binding loop of LET‐60/Ras—revealing a Ras > PI3K axis that restrains somatic growth, contrary to canonical IIS predictions.

Together, these results demonstrate how structural modeling can inform genome editing to produce precise, interpretable alleles that probe conserved signaling mechanisms in vivo. More broadly, this approach bridges molecular structure and organismal physiology, providing a framework for understanding how defined PI3K perturbations influence aging, metabolism, and developmental plasticity.

## 2 Results

### 2.1 Endogenous AGE‐1/PI3Kcat Is Expressed Ubiquitously With No Evident Subcellular Localization

**FIGURE 1:**
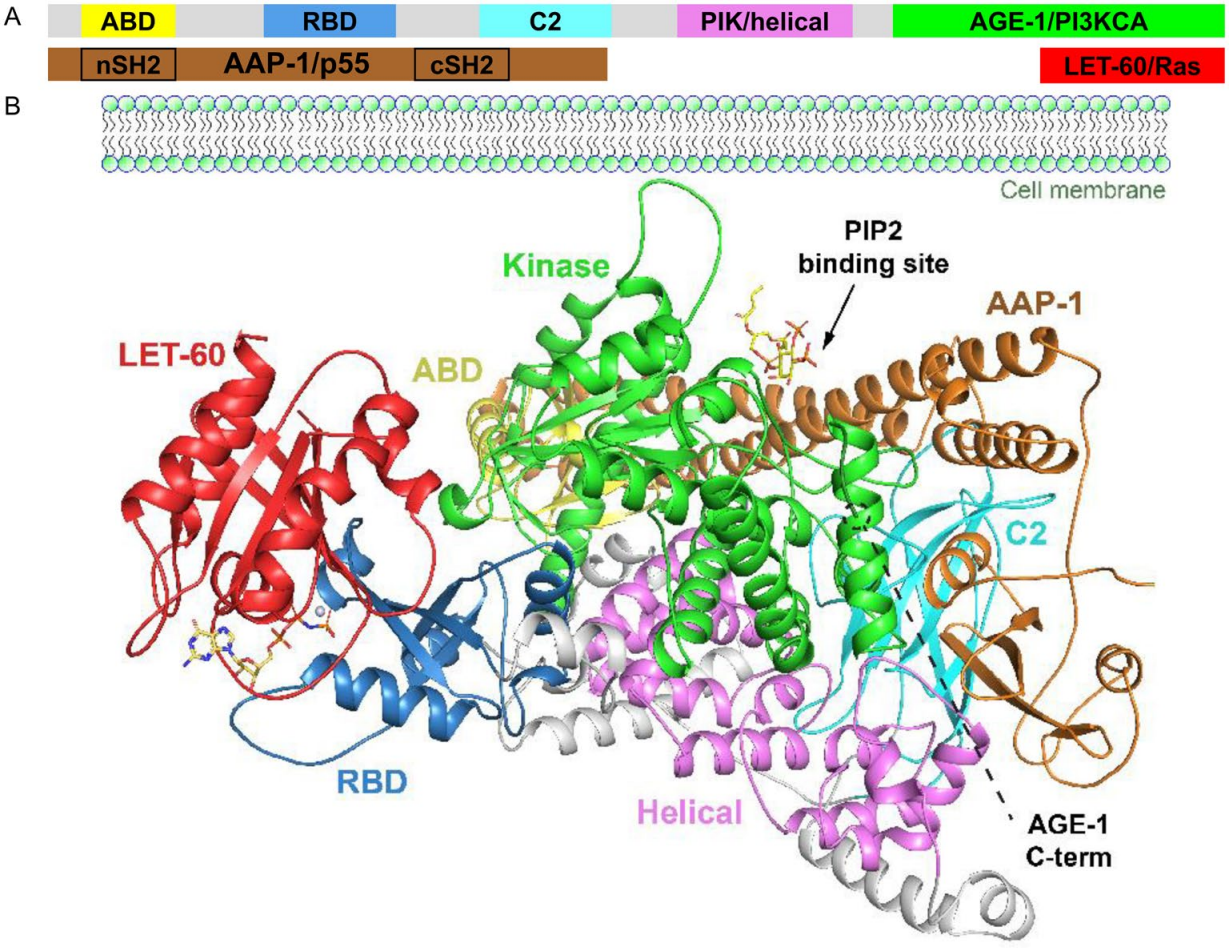
A structural model of the Ras—PI 3‐Kinase signaling complex. (A) Conserved domains diagram of AGE‐1/PI3Kcat, AAP‐1/p55/PI3Kreg and LET‐60/Ras small GTPase. Domains from left to right: ABD = adaptor binding domain; RBD = Ras binding domain; C2 = PI(4,5)P_2_ binding; PIK/helical = conserved in PI3Ks and PI4Ks; PI3KCA = catalytic lipid kinase domain; SH2 = phosphotyrosine binding domain associated with binding p‐Tyr in signaling cascades. AGE‐1: AGEing alteration. Catalytic subunit of PI3K (PI3KCA). AAP‐1: AdAPtor for PI3K. p55/p85 in mammals. Note that *C. elegans* AAP‐1 does not encode an identifiable inter‐SH2 sequence (iSH2) domain found in mammalian p50/p55 regulatory subunits, but a coiled‐coil is detected in the central region, which like substitutes for the same activity and the structural level. LET‐60, LEThal; The Ras small GTPase orthologous to human H,N,K‐Ras. (B) A *post hoc* structural model of the predicted *C. elegans* AGE‐1 PI3KCA catalytic subunit, a portion of the AAP‐1 ortholog of the human p50/p55 regulatory subunit (not including cSH2, which is also absent for various mammalian structures) and LET‐60 with Mg^++^ and GMPPNP, a non‐hydrolysable GTP analog. The plasma membrane is oriented upwards in the diagram; accordingly, the C‐terminal end of LET‐60 ⍺5 is oriented upwards. (The C‐terminal hypervariable region+CAAX is truncated in most structures since the sequence is unstructured and lipid modified. But the HVR is expected to abut the PM and the CAAX (Cys‐Ali‐Ali‐X, where Ali = aliphatic and X = any residue) is modified with a farnesyl lipid and proteolytically processed.) The putative PI(4,5)P_2_ binding sequence and phosphoinositol headgroup, shown, is also upwardly oriented toward the expected location of the PM. See Methods for assembly of this structural model. This C‐term is indicated, located far enough from the PM to potentially avoid steric conflict with the association of the enzyme complex with the PM.

To visualize the expression of AGE‐1 in live animals, we used CRISPR/Cas9‐dependent genome editing to insert sequences encoding mNeonGreen fluorescent protein (mNG) and 2xHA epitope tag into the endogenous *age‐1* gene. First, threading of the *C. elegans* sequence of AGE‐1/PI3Kcat, particularly the positioning of the C‐term ⍺‐helix 5 (⍺5) of LET‐60, the N‐term of AGE‐1, and the positioning of the PIP2 head group (Figure 1A,B; S1A,B), suggest that an N‐terminal tag may interfere with the association of AGE‐1 with the plasma membrane. Second, we observed an N‐terminal extension in *C. elegans* AGE‐1: 57 residues longer than human PIK3CA and 38 residues longer than *Drosophila* PI3K92E. This AGE‐1 extension contains seven Arginine residues relative to one in *Drosophila* (Figure S1). Based on location in the protein relative to the plasma membrane and established mechanisms of proteins associating with the plasma membrane, this extension may constitute an electrostatic interaction between the N‐terminus of AGE‐1 and acidic microdomains in the plasma membrane that is not present in orthologous proteins, thus counter‐indicating an N‐terminal tag. Third, C‐terminal tagging may sterically hinder AGE‐1 protein function. Publication of structures of human PI3Kα and KRAS during preparation of this manuscript corroborated these observations (Czyzyk et al. 2025; Torosyan et al. 2025).

**FIGURE 2:**
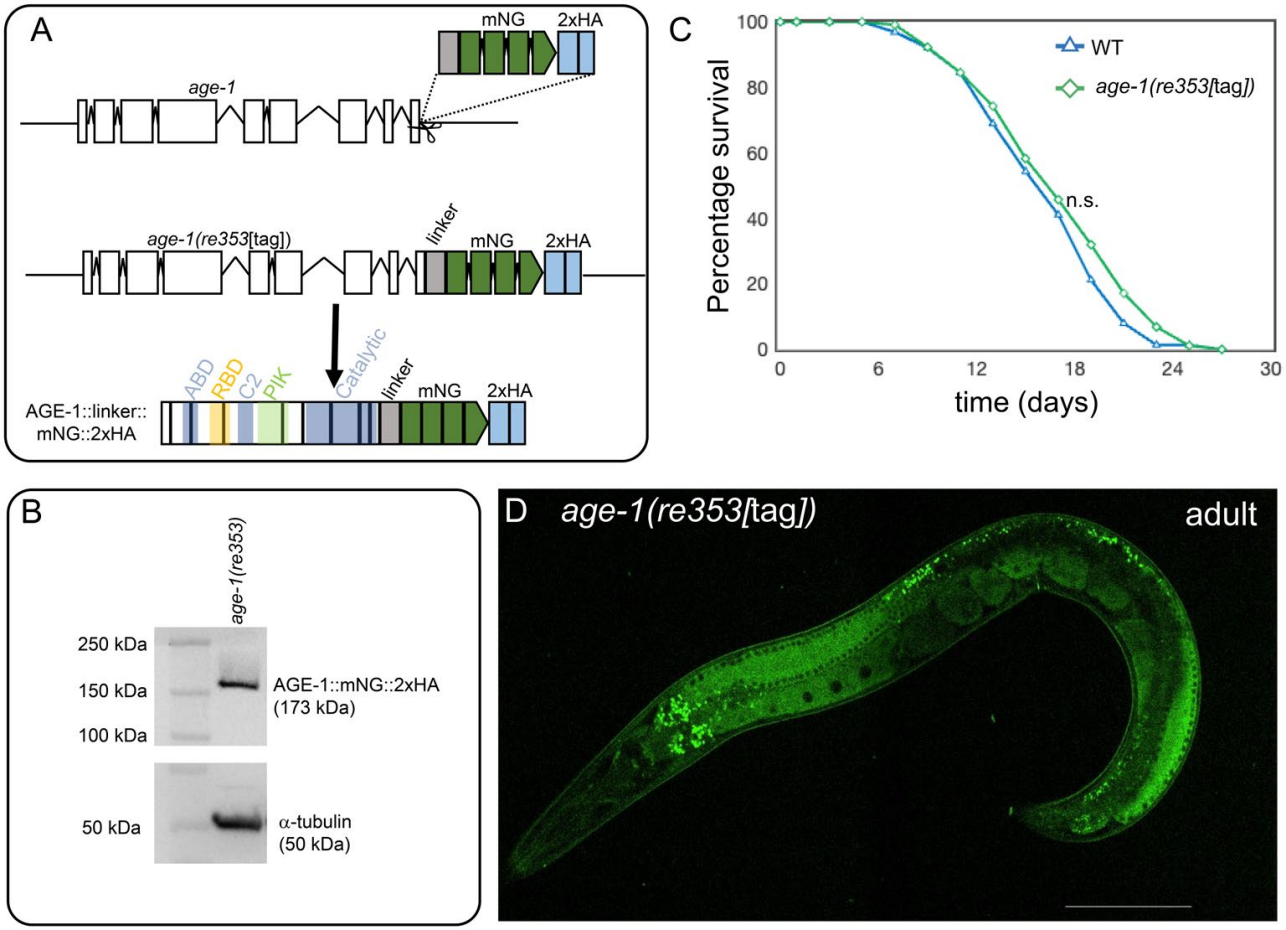
Tagging endogenous AGE‐1 at the C‐terminus. (A) A schematic of the *age‐1* gene and 3′‐end insertion strategy for a C‐terminally tagged endogenous protein. The exon‐intron gene, defined by extensive RNAseq data, is shown at the top with the PCR product repair template used, generated with 35 bp homology arms at each end to support homology‐directed repair (HDR). The resulting construct (middle) was detected and tracked by triplex PCR and confirmed by Sanger sequencing of all novel and flanking sequences. The bottom model shown the predicted resulting protein; colors indicate different functional domains. Lines indicate different exons. (B) Immunoblotting of edited *age‐1(re*353[tag]*)* animals with ⍺‐HA antibody revealed the predicted 173 kDA protein predicted by AGE‐1 + tag sequences, with α‐tubulin control. (C) Kaplan–Meier curves show percent survival of wild‐type animals versus *age‐1(re353*[tag]) animals, analyzed concurrently, with no difference observed. *N* = sample size. *p*‐value was calculated by Log‐rank Mantel‐Cox test. n.s. = no significant difference. (D) A spinning disk confocal photomicrograph (488 nm) of an optical section of an *age‐1(re*353[tag]*)* adult animal, showing likely ubiquitous expression. Left = anterior, down = dorsal. Dark circles are nuclei, particularly standing out in oocytes in the proximal gonad and small nuclei in the distal gonad. Bright punctae are intestinal autofluorescence of gut granules, which are lipid storage depots. Additional images are shown in (Figure S2). Variability of intensity depends on position in the low‐magnification field with variable excitation. Scale bar is 100 μm.

Consequently, to minimize the likelihood of perturbing AGE‐1 function, we inserted sequences encoding a 30‐residue linker at the 3′ end of *age‐1* but 5′ to the sequences encoding mNG::2xHA (Figure 2A). The resulting *age‐1(re353[age‐1::linker::mNG::2xHA])* mutant, here referred to as the *age‐1(re353[tag])* or *age‐1(*tag*)* mutant animal, was detected by triplex PCR—two flanking primers and one primer within the edit—and validated by sequencing (see Methods and see Table S2 for oligonucleotide sequences).

After western blotting, immunodetection using anti‐HA antibodies revealed a band in *age‐1(re353[*tag*])* animals consistent with the 173 kDa expected from AGE‐1::linker::mNG::2xHA (Figure 2B). To assay the impact of tagging on the function of AGE‐1, we assayed the lifespan of *age‐1(re353[tag])* animals vs. the wild type. The two lifespans were not significantly different (Figure 2C).

We surveyed expression of AGE‐1::linker::mNG::2xHA in *age‐1(re353[*tag*])* animals using spinning disk confocal microscopy. Expression was ubiquitous in the animal with potentially elevated levels in the germline (Figure 2D). Expression was cytoplasmic, as evinced by nuclear exclusion of AGE‐1. Localization was also consistent throughout developmental stages, including embryos (Figure S2A–D). We did not observe evidence of subcellular localization, though we only examined this phenomenon closely in the vulval precursor cells (Figure S2C), when a role for AGE‐1 signaling had been hypothesized (see below). Thus, we observed that AGE‐1/PI3Kcat is expressed globally, with each cell throughout the life of the animal potentially capable of responding to upstream signals to activate AGE‐1 activity.

### 2.2 E630K Mutant Endogenous AGE‐1/PI3Kcat Decreases Lifespan Consistent With Established Roles of Other IIS Components

**FIGURE 3:**
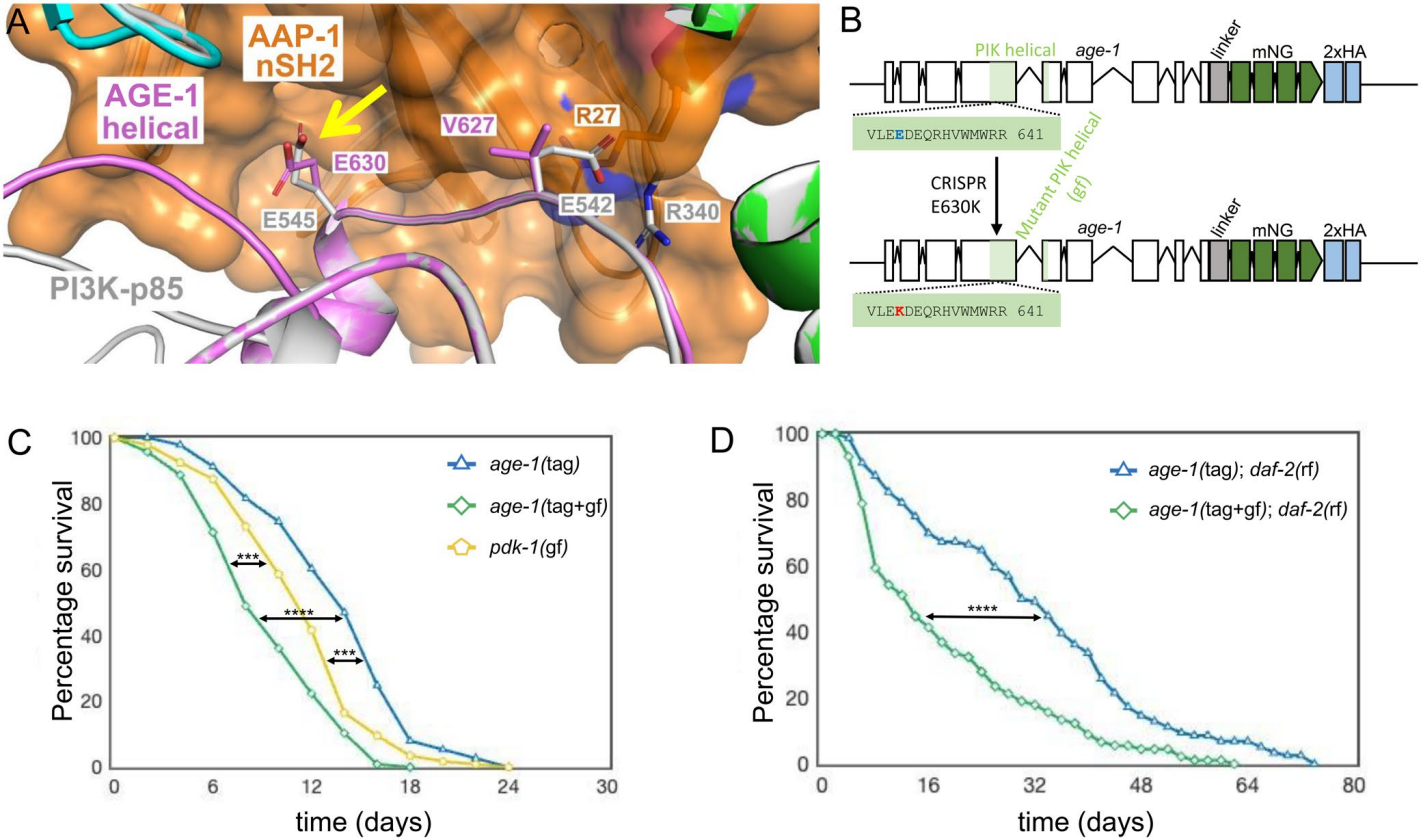
The *C. elegans* E630K equivalent to human oncogenic mutation E545K causes a gain of function. (A) A zoom of a structural model of the inhibitory interaction of the helical/PIK domain of PI3K catalytic subunit with the nSH2 domain of the regulatory subunit (pink and brown, as in Figure 1B). Threaded AGE‐1‐AAP‐1 structure is overlaid with the PI3Kcat‐p85 structure, human‐nematode. The yellow arrow indicates the overlay depicts of human E545 (gray) and nematode E630 (pink). Solid pink indicates predicted AGE‐1, solid gray depicts solid human PI3K, mottled pink/gray indicates predicted co‐positioning of the two polypeptides. While the E545/E630 residues align, the human E542, also commonly mutated to a K in human cancers, aligns with nematode V627 (see sequence alignments in Figure S3A,B). (B) A schematic of *age‐1(re353[age‐1::Linker::mNG::2xHA])* with the wild‐type E630 residue above in blue and the putative gain‐of‐function E630K mutation below in red, encoded by *age‐1(re353re392*gf[tag E630K]*)*. (C) A Kaplan–Meier curve showing that *age‐1(*tag+gf*)* and *pdk‐1(mg142*gf*)* gain‐of‐function mutants for the PI3K>PDK>Akt pathway live shorter than *age‐1(*tag*)* animals, as predicted by previous genetic analysis. Arrows indicate *p* values. *N* = 140 (36 censored) for *age‐1(*tag*)*. *N* = 140 (20 censored) for *age‐1(*tag+gf*)*. *N* = 140 (22 censored for *pdk‐1(mg142*gf*)*). (D) The activating mutant *age‐1(*tag+gf*)* also strongly reverses the longevity conferred by reduced function of InsR *daf‐2(e1370*rf*)* double with *age‐1(*tag), thus with only the E630K missense mutation different between the strains. *N* = 140 (40 censored) for *age‐1(*tag*); daf‐2(e1370*rf), *N* = 160 (44 censored) for *age‐1(*tag+gf*); daf‐2(e1370*rf). *p* values calculated by Kaplan–Meier estimator, Log‐rank (Mantel‐Cox) test. For *p* values, *≤ 0.05, **≤ 0.01, ***≤ 0.001, ****≤ 0.0001.

From a very large cohort, 12.1% of tumors harbor activating missense mutations in human PI3Kcat/p110α, encoded by PI3KCA (Sivakumar et al. 2023; Zhao and Vogt 2008). These cluster in a hotspot in the helical/PIK domain N‐terminal to the lipid kinase domain (Figure 3B; S3A,B). These mutations alter either of the first two glutamates (Es), E542 or E545, of the four acidic residues in the “EITEQEKD” sequence, changing them to lysines (K) in a charge reversal. E542 and E545 oncogenic mutations disrupt the inhibitory salt‐bridge interaction between the helical domain of PI3Kcat and the nSH2 domain of the p55/p85 regulatory subunit of PI3K (Carson et al. 2008; Miled et al. 2007; Zhao and Vogt 2008). Consequently, these mutations increase lipid kinase activity, elevate levels of PIP_3_, which in turn recruits PDK and Akt to the plasma membrane via their PIP_3_‐binding PH domains, and increase levels of downstream phospho‐Akt and phospho‐S6K (Carson et al. 2008; Currie et al. 1999; Gymnopoulos et al. 2007; Ikenoue et al. 2005). Alignment with *Drosophila* and *C. elegans* AGE‐1/PI3Kcat orthologs with human PI3Kα reveals four acidic residues in this interval in AGE‐1 and human PI3Kcat/p110α (Figure 3B; S3A,B). In *C. elegans* this sequence is clustered as “VLEEDEQ”. Threading *C. elegans* sequences onto the mammalian structure implies the same structural positioning of *C. elegans* E630 and human E545 (Figure 3A). Thus, E630K in *C. elegans* AGE‐1 likely has the same effect as E545K in PI3Kcat.

To introduce a potentially constitutively activating mutation into our endogenous AGE‐1::linker::mNG::2xHA, we used CRISPR/Cas9‐dependent genome editing to alter E630 to K, thereby generating *age‐1(re353re392*gf*[*tag E630K*])*, hereafter *age‐1(*tag+gf*)*. Immunoblotting with anti‐HA antibody revealed no change in stability in the tagged E630K AGE‐1 protein relative to *age‐1(re353[tag])* alone (Figure S3C), and the E630K gf mutation does not overtly alter fluorescence levels or protein localization (Figure S3E,F).

The *age‐1(hx546)* reduction‐of‐function mutation in *C. elegans* PI3Kcat was the first lifespan‐extending mutation found in animals (Friedman and Johnson 1988a, 1988b; Johnson 1990; Klass 1983). We reasoned that a constitutively activating, gain‐of‐function mutation in AGE‐1 would reduce lifespan, consistent with gain‐of‐function mutations in PDK‐1 and AKT‐1 (Paradis et al. 1999; Paradis and Ruvkun 1998) and loss of inhibitory DAF‐18/PTEN lipid phosphatase function (Ogg and Ruvkun 1998). We measured the lifespan of *age‐1(*tag+gf*)* animals compared to that of *age‐1(*tag*)* animals and the published gain‐of‐function *pdk‐1(mg142*gf*)* mutant animals (Paradis et al. 1999). As expected, *pdk‐1(mg142*gf*)* animals lived shorter than *age‐1(*tag*)* animals, which lived a wild‐type span (Figure 3C). *age‐1(*tag+gf*)* animals lived shorter than not only *age‐1(*tag*)* animals but also *pdk‐1(mg142*gf*)* animals (Figure 3C). The *age‐1(*tag+gf*)* mutation also reversed the longevity conferred by mutation of DAF‐2/InsR, *daf‐2(e1370)* (Figure 3D). This epistatic interaction is consistent with the established biochemical role of PI3K downstream of Insulin/IGF receptors in signal transduction (Dorman et al. 1995). We also generated tag‐free *age‐1(re489[*E630K*])*, hereafter *age‐1(*gf*)*, and observed that it decreased lifespan relative to the wild type (Figure S3D). These results are consistent with the interpretation that E630K increases endogenous AGE‐1/PI3Kcat signaling in vivo.

### 2.3 E630K Mutant Endogenous AGE‐1/PI3Kcat Reverses a Biomarker of Deficient DAF‐2/InsR

The DAF‐16 ortholog of mammalian FOXO1,3,4,6 transcription factors has been shown genetically to be inhibited by IIS signaling: the DAF‐2 > AGE‐1 > PDK‐1 > AKT‐1/2 cascade phosphorylates and inhibits DAF‐16/FOXO, mirroring the mammalian cascade (Brunet et al. 1999; Dorman et al. 1995; Ogg et al. 1997). GFP‐tagged DAF‐16 has been validated as a reporter for DAF‐2/InsR pathway activation; reduced DAF‐2/IIS function induces nuclear translocation of DAF‐16::GFP transgenes (Demirbas et al. 2025; Henderson and Johnson 2001; Lee et al. 2001).

**FIGURE 4:**
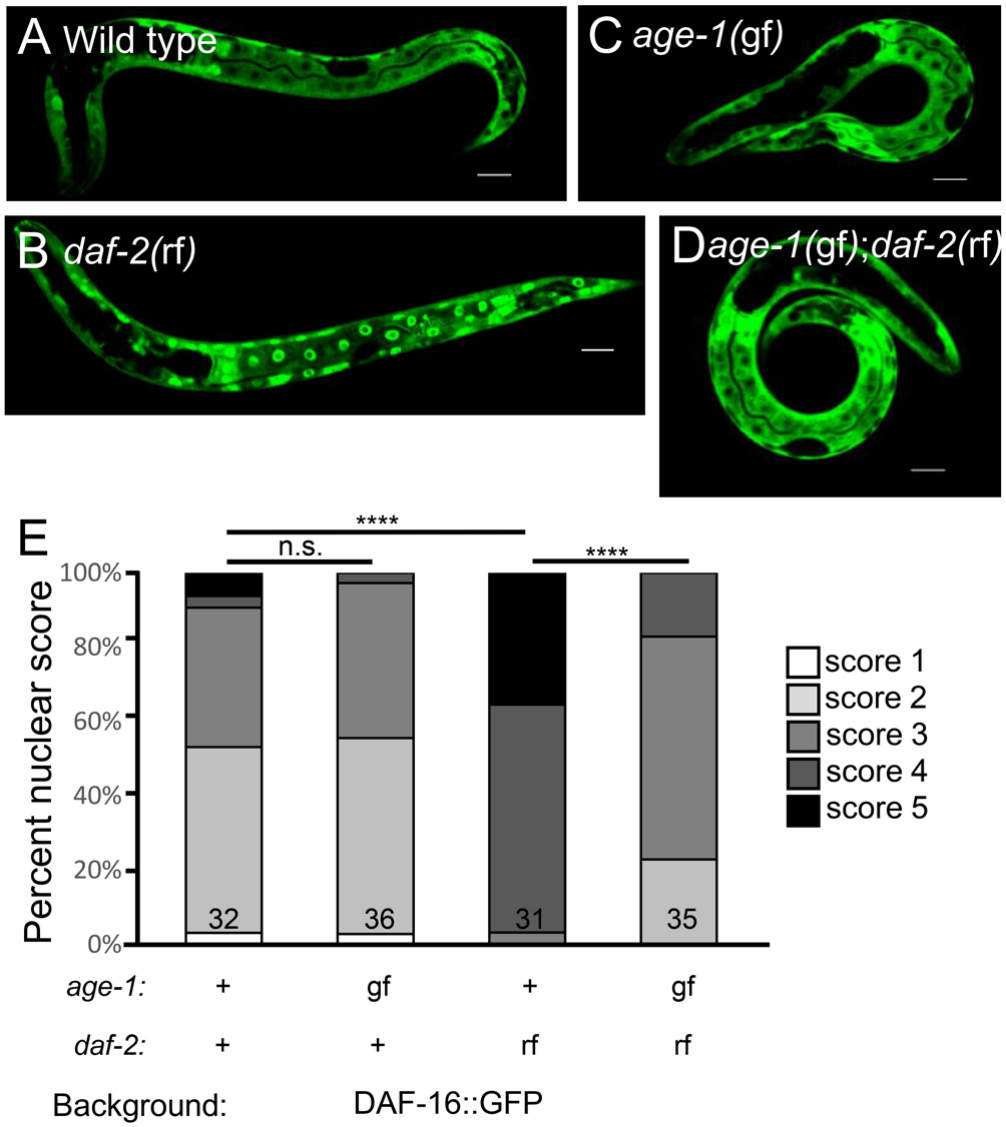
E630K mutant AGE‐1 reverses deficient DAF‐2‐dependent nuclear translocation of DAF‐16::GFP. (A–D) Confocal photomicrographs of DAF‐16::GFP transgenic animals (*zIs356[daf‐16p>daf‐16a,b::GFP+rol‐6(su1006*d*)]*) with (A) wild type, (B) *daf‐2(m577rf)*, (C) *age‐1*(E630K), and (D) *age‐1(*gf*)*; *daf‐2(m577*rf*)* genotypes. (E) Quantification of blind scoring of nuclear localization of DAF‐16::GFP with various genotypes. Scoring rubric: 1 = some nuclear exclusion (not nucleolus based on larger size of black spot); 2 = no difference between nucleus and cytoplasm; 3 = slightly higher nucleus than cytoplasm; 4 = stronger nuclear than cytoplasm; 5 = very strong nuclear translocation. *p* values were calculated by the Mann–Whitney test, ****≤ 0.0001. Scale bars = 50 μm.

We used the *zIs356[daf‐16p>daf‐16a/b::GFP+rol‐6(su1006*d*)]* transgene (Henderson and Johnson 2001), hereafter DAF‐16::GFP, as a biomarker to assess the impact of the E630K AGE‐1 mutation on IIS pathway output. As described, reduction of DAF‐2/InsR function induced nuclear translocation of DAF‐16::GFP (Figure 4A,B). The *age‐1(*E630K*)* mutant—untagged—suppressed the *daf‐2(*rf*)* phenotype, reversing nuclear localization of DAF‐16/FOXO (Figure 4C,D) and reducing heat shock‐induced nuclear localization of DAF‐16::GFP (Figure S4A). This epistatic interaction in control of DAF‐16::GFP nuclear translocation provides further evidence that E630K increases activity of the endogenous AGE‐1/PI3Kcat pathway.

### 2.4 E630K Mutant Endogenous AGE‐1/PI3Kcat Increases Induction of 1° Vulval Precursor Cell Fate

**FIGURE 5:**
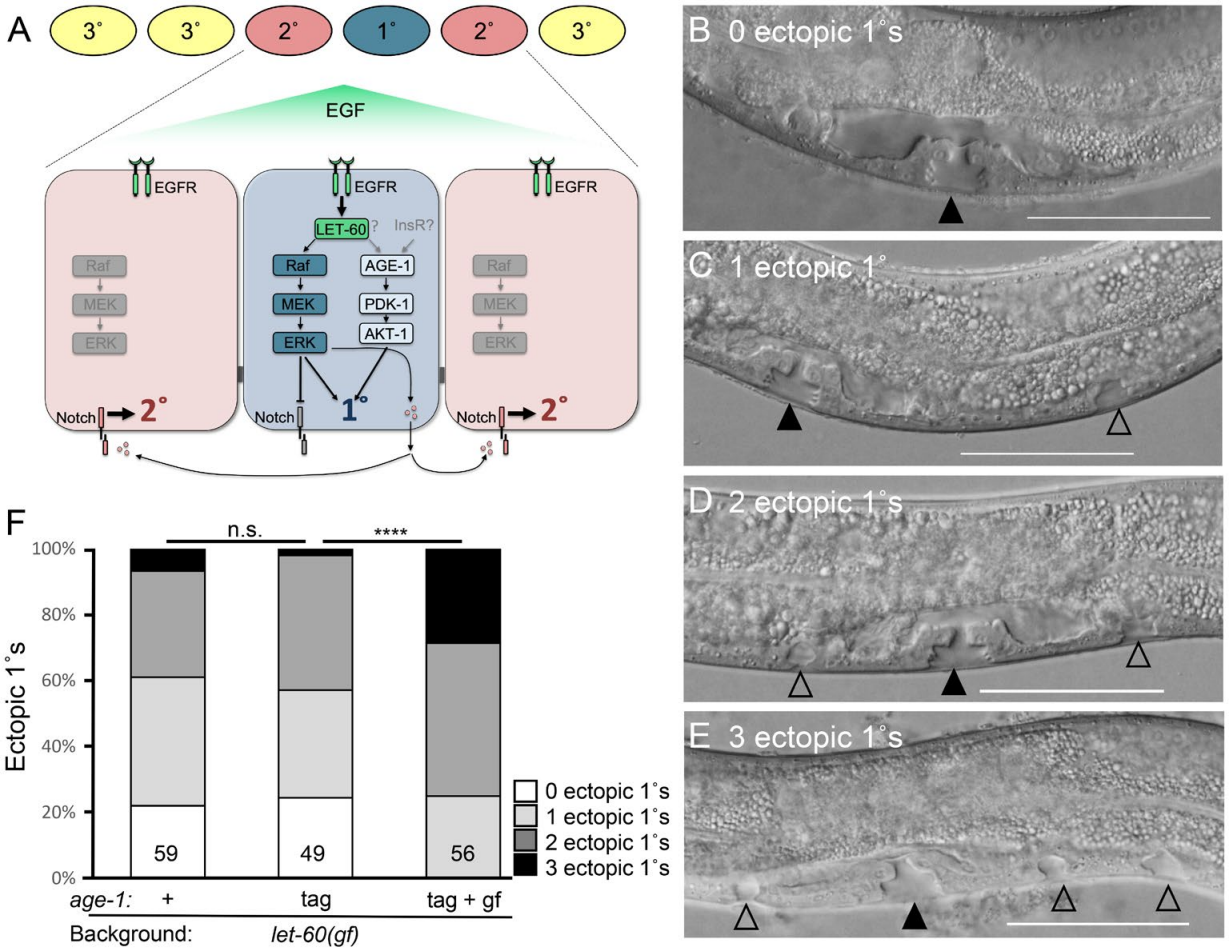
Constitutive activation of AGE‐1/PI3Kcat increases induction of 1° cell fates among VPCs. (A) A schematic of developmental patterning of the vulval precursor cells (VPCs). EGF signaling induces 1° fate via the necessary and sufficient EGFR>Ras>Raf>MEK>ERK/MAP kinase pathway. PDK‐>AKT‐1 promotes 1° fate as a modulatory pathway, with inhibition by DAF‐18/PTEN lipid phosphatase implying a potential 1°‐promoting role of AGE‐1/PI3Kcat. The source of activating signal, DAF‐2/InsR, or LET‐60/Ras, is unknown. (B–E) Photomicrographs of DIC imaging of late 4th larval stage (L4) animals with zero (B) to three (E) ectopic 1° lineages. Black arrowheads indicate the normal morphogenetic vulva, formed of 2°‐1°‐2° lineages. Open arrowheads indicate ectopic pseudovulvae, formed from inappropriately induced 1° lineages. Genotypes are in black. *let‐60(n1046*gf*)* is a G13E mutant that moderately induces ectopic 1° cells and is sensitive to increases or decreases to 1°‐promoting signaling. Ventral is down and anterior is left. Scale bars = 50 μm. (F) Quantification of ectopic 1° pseudovulvae from each genotype. *N* is indicated in the bar for each genotype. *p* value was calculated by the Mann–Whitney test, **** ≤ 0.0001.

In response to a point source of epithelial growth factor (EGF) from the ventral gonad, the initially equipotent *C. elegans* vulval precursor cells (VPCs) are induced to form the 3°‐3°‐2°‐1°‐2°‐3° pattern of cell fates with 99.8% accuracy (Braendle and Felix 2008; Shin et al. 2019; Figure 5A). The necessary and sufficient signaling cascades that direct this developmental event are the EGFR>Ras>Raf>MEK>ERK canonical MAP kinase pathway promoting 1° cell fate and the DSL>Notch receptor>CSL pathway promoting 2° cell fate (Shin et al. 2018). Additional modulatory pathways also contribute to this process: the LET‐23/EGFR>LET‐60>RGL‐1>RAL‐1>GCK‐2 cascade promotes 2° cell fate in support of the Notch pathway (Shin et al. 2018, 2019) while PDK‐1/PDK>AKT‐1/Akt promotes 1° fate in support of the canonical ERK/MAP kinase pathway (3° fate is promoted by an independent Rap2>MAP4K signal (Fakieh and Reiner 2025)). An inhibitory role for DAF‐18/PTEN has been described for the 1°‐promoting modulatory signal (Nakdimon et al. 2012; Shin et al. 2019). Thus, gain of AGE‐1/PI3Kcat function would be expected to phenocopy loss of DAF‐18/PTEN and promote 1° induction.

The VPC patterning system is significantly buffered against extraneous developmental noise (Braendle and Felix 2008; Shin et al. 2019). Given this buffering and the parallel operation of multiple signaling cascades, functions of modulatory pathways are most sensitively resolved in a genetic background harboring the modestly constitutively activating G13E mutation in the *C. elegans* Ras ortholog, *let‐60(n1046*gf*)*, by quantifying increases or decreases in ectopic 1° VPC fates (Figure 5A–E). This assay provides a sensitive and reproducible readout of the effect of modulatory signaling cascades on VPC fate patterning. This approach has been used broadly to interrogate the network of signaling pathways governing VPC fate specification (Berset et al. 2001; Berset et al. 2005; Fakieh and Reiner 2025; Shin et al. 2019; Shin et al. 2018; Yoo et al. 2004; Yoo and Greenwald 2005; Zand et al. 2011) reviewed in (Shin and Reiner 2018).

AGE‐1::linker::mNG::2xHA, encoded by *age‐1(*tag*)*, did not alter the induction of ectopic 1° pseudovulvae by *let‐60(n1046*gf*)* compared to wild type (Figure 5F; S5A). This observation is consistent with prior validation that the *age‐1(tag*) allele does not measurably perturb pathway output. In contrast, *age‐1(*tag+gf*)* strongly increased induction of ectopic 1° pseudovulvae (Figure 5F; S5A), thus phenocopying loss of DAF‐18/PTEN and gain of PDK‐1 and AKT‐1 functions (Nakdimon et al. 2012; Shin et al. 2019).

Taken together, our results with the E630K mutant AGE‐1(tag+gf) allele—reducing lifespan, reversing DAF‐16::GFP nuclear translocation in *daf‐2(*rf*)* animals, and promoting ectopic 1° fate induction—support the conclusion that E630K increases endogenous AGE‐1/PI3Kcat signaling in vivo. These results validate our structure‐guided approach for engineering endogenous proteins to generate alleles that phenocopy mutations characterized in mammalian systems.

### 2.5 Mutations in the LET‐60:AGE‐1/PI3Kcat Binding Interface Reveal a Role in Restricting Animal Stature

The PI3K catalytic subunit can be activated through two general mechanisms: (i) recruitment of PI3Kcat to phosphotyrosines on receptor tyrosine kinases at the plasma membrane via the p55/p85 regulatory subunit, or (ii) engagement of GTP‐bound Ras with the Ras‐binding domain (RBD) of PI3Kcat, promoting membrane association and activation (Cuesta et al. 2021). A single mutation, *age‐1(ag12)*, introducing an L336F change in the RBD of AGE‐1 (L267F in human PI3KCA), was isolated previously in a mutant screen for increased resistance to infection by the pathogen *Pseudomonas aeruginosa* (Miyata et al. 2008). This L336F change of *age‐1(ag12)* also increased animal lifespan and at 25°C caused formation of dauer rather than L3 larvae, a diapause arrested alternative third larval stage whose induction is frequently associated with low IIS activity. Moreover, this L336F mutant residue lies in the hydrophobic interior of the RBD domain; the increased size of the L336F side chain may thus disrupt hydrophobic packing of the interior and potentially destabilize the RBD or the entire AGE‐1 protein. Consequently, the L336F mutation may not specifically disrupt binding of LET‐60/Ras to the RBD of AGE‐1. A mutant strain containing the *age‐1(ag12)* could not be recovered from the original strain collection.

**FIGURE 6:**
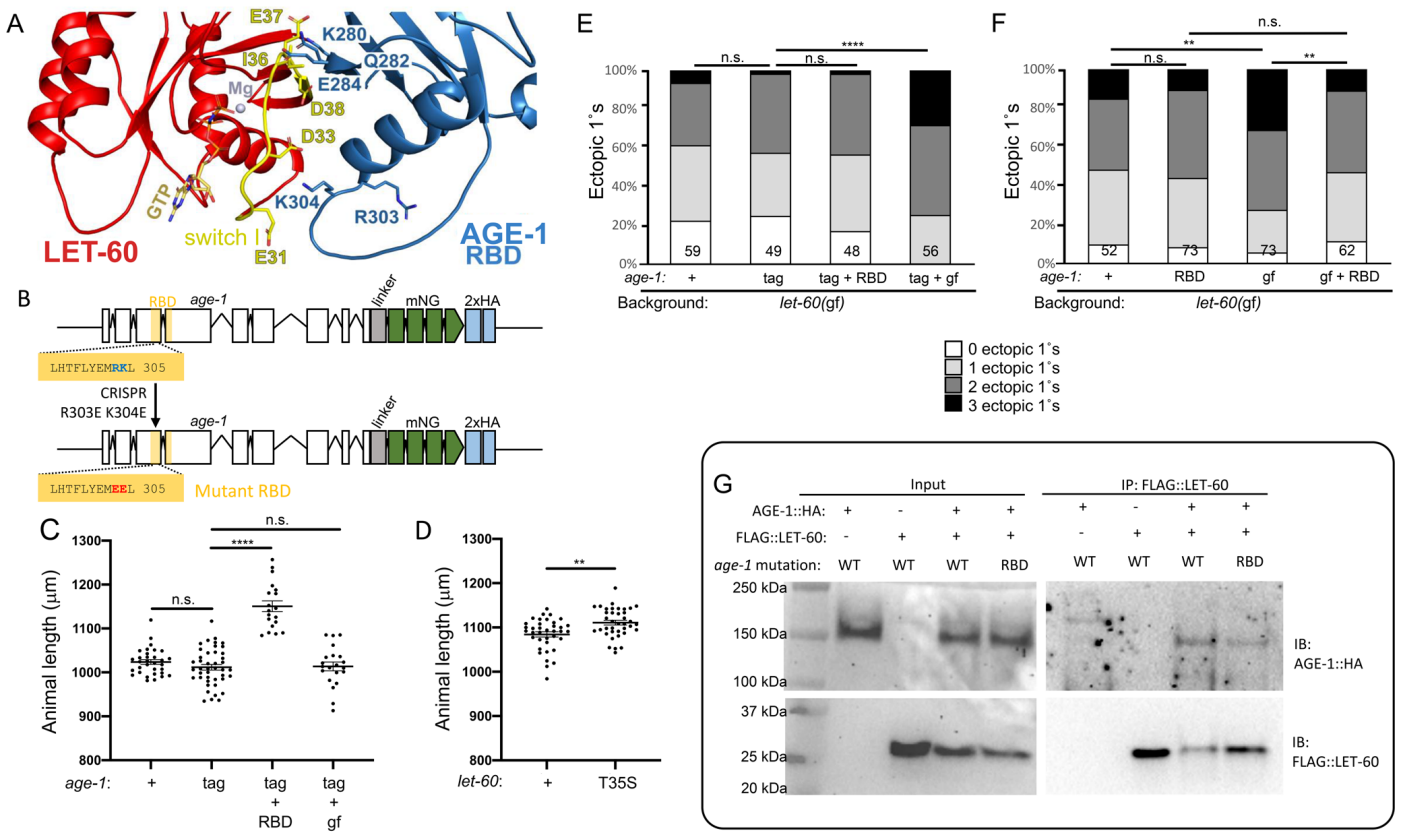
Defective association of LET‐60/Ras with AGE‐1/PI3Kcat reveals a role in constraining body stature. (A) A zoomed portion of the same structural model as in Figure 1B of LET‐60/Ras and the RBD of AGE‐1/PI3Kcat. K304 of the AGE‐1 RBD may interact with Switch I of LET‐60/Ras, such that replacement with the charge‐reversing side chain with Glu (E) may repel LET‐60 and reduce binding. R303 faces the neighboring loop, such that replacement with the charge‐reversing side chain with Glu (E) may interfere with positioning of the interface of the RBD. Also shown are K280, Q282, and E284 which are also predicted to disrupt the interface with LET‐60 if mutated to E, A, and K, respectively, in some combination. But the sgRNA for CRISPR for R303 and K304 was predicted to be more robust, so we mutated that site first and were successful. (B) A schematic of *age‐1(re353[age‐1::Linker::MNG::2xHA])* with the wild‐type R303 K304 residues above in blue and the R303E K304E putative interface disrupting mutation below in red, encoded by *age‐1(re353re377RBD*[tag R303E K304E]*)*. (C) Animal length in microns as measured from DIC photomicrographs for RBD and activating mutations in AGE‐1. (D) Animal length in microns as measured from DIC photomicrographs for the LET‐60 T35S putative Raf‐selective mutant vs. wild type. Animals in each panel for C and D were scored concurrently, but the experiments presented in C and D were scored at widely separated time points. (E) Quantification of ectopic 1° pseudovulvae in the *let‐60(n1046*gf*)* background reveals no role of the RBD in 1° fate induction. (F) The same *age‐1* edits were tested in the absence of tag, with the same findings for RBD and gf. But in the RBD+gf double mutant, RBD is epistatic to gf. (G) Anti‐flag immunoprecipitation of 3xFLAG::D10::LET‐60 co‐immunoprecipitated AGE‐1::Linker::2xHA, which was reduced in AGE‐1:::Linker::2xHA(RBD). n.s. = not significant, **≤ 0.01, ****≤ 0.0001, *p* value was calculated by the *t*‐test (C, D) and Mann Whitney test (E, F).

Consequently, we undertook a structure‐directed approach to disrupt the binding interface of LET‐60/Ras and the RBD of AGE‐1. The core effector binding loop of LET‐60/Ras is 100% conserved among model invertebrates and mammals, presumably reflecting the evolutionary constraints of a single short polypeptide required to interact with several effector proteins (Reiner and Lundquist 2018). In contrast, the primary sequence of the AGE‐1 RBD is not well conserved relative to mammalian PI3K catalytic subunits (Figure S6A,B). We used structural models to infer the importance of two basic residues, Arg‐303 and Lys‐304, predicted to contribute to and lie at the binding interface between LET‐60/Ras and the AGE‐1/PI3Kcat RBD (Figure 6A,B).

We used CRISPR/Cas9‐dependent genome editing to substitute codons encoding acidic glutamate (E) residues for the wild‐type basic Arg‐303 and Lys‐304 codons in *age‐1(*tag*)*, resulting in the *age‐1(re353re377*rf*[age‐1::mNG::2xHA(*tag R303E, K304E*)])* mutant animal, hereafter *age‐1(*tag+RBD*)*. The RBD mutations did not destabilize AGE‐1::linker::mNG::2xHA protein as detected by immunoblotting for HA (Figure S6C) nor alter expression levels or localization of AGE‐1 (Figure S6E,F).

We tested whether LET‐60 and AGE‐1 interact physically in vivo by co‐immunoprecipitation of tagged endogenous proteins. Accordingly, we generated tagged LET‐60 by serially introducing sequences encoding d10 and then 3xFLAG into the 5′ end of endogenous *let‐60* (Figure S6D; see Methods). These edits were validated by immunoblotting using anti‐human RAS antibody raised to an epitope that is 100% identical between nematodes and humans (Figure S6G).

In double edited AGE‐1::HA, FLAG::LET‐60 animals, immunoprecipitation (IP) of FLAG::LET‐60 yielded detectable AGE‐1::HA (Figure 6G). IP of FLAG::LET‐60 with AGE‐1(tag+RBD) reduced the AGE‐1 band. Repeat IPs from the same lysates yielded similar results (Figure S6H). The co‐IP signal was modest, consistent with a small fraction of endogenous LET‐60 and AGE‐1 molecules existing in complex at any given time, potentially reflecting developmental, cell‐specific, or environmental regulation. These data support the interpretation that the R303E, K304E substitutions reduce LET‐60:AGE‐1 interaction in vivo.

Unexpectedly, in *age‐1(*tag+RBD*)* mutants we observed that animals were larger than stage‐matched wild‐type or *age‐1(*tag*)* controls. This was true for both animal length (Figure 6C) and width (Figure S6I). The RBD mutations did not reduce induction of 1° cell fate in the VPC system (Figure 6E), nor did the RBD mutations in untagged *age‐1* alter 1° ectopic induction (Figure 6F), consistent with LET‐60/Ras not measurably activating AGE‐1 in VPC fate patterning under assay conditions. The RBD mutations also did not alter lifespan (Figure S6K,L), in contrast to the pleiotropic phenotypes associated with *age‐1(ag12)*, which likely reflects broader destabilization of AGE‐1. Thus, we hypothesize that these RBD substitutions selectively perturb the LET‐60/Ras:AGE‐1 interface without broadly impairing AGE‐1 function, revealing a previously unrecognized role for this interaction in constraining organismal growth.

### 2.6 The T35S Putative Raf‐Selective Mutation in LET‐60/Ras Phenocopies the Putative RBD‐Deficient Mutation in AGE‐1/PI3Kcat

To further test whether LET‐60/Ras binding to AGE‐1/PI3Kact regulates body stature, we selectively mutated the effector binding loop (EBL) of endogenous LET‐60/Ras to reduce binding to AGE‐1/PI3Kact. A series of effector binding mutations were previously defined via yeast two‐hybrid analysis and cell‐based studies that selectively supported activation of certain proto‐oncogenic effectors by constitutively activated Ras (Rodriguez‐Viciana et al. 1997; White et al. 1995; Wolthuis and Bos 1999). The efficacy of this mutation was validated via structural biology and in vitro binding (Pacold et al. 2000). Of the canonical EBL mutants, T35S is Raf‐selective, E37G is RalGEF‐selective, and Y40C is PI3Kcat‐selective. However, these mutations do not necessarily fully cleanly or completely interfere with function: they may not retain 100% activation of effectors nor specifically block one effector.

We previously used constitutively activated LET‐60 G12V,T35S and G12V,E37G via VPC‐specific transgenesis; both functioned in vivo as expected from the mammalian literature: G12V,T35S appeared to activate the LET‐60/Ras>LIN‐45/Raf 1°‐promoting pathway and G12V,E37G appeared to activate the LET‐60/Ras>RGL‐1/RalGEF 2°‐promoting pathway (Zand et al. 2011). However, LET‐60/Ras activation of LIN‐45/Raf is essential for viability in *C. elegans* (Yochem et al. 1997). Consequently, we cannot use mutations other than the T35S EBL mutation in endogenous LET‐60/Ras because of the expectation they will eliminate activation of LIN‐45/Raf and hence result in lethality. Thus, we are constrained to using the T35S mutation in endogenous LET‐60/Ras to impair LET‐60 binding to AGE‐1. This mutation is also expected to reduce RalGEF signaling, as well as perhaps other, non‐proto‐oncogenic effectors.

We used CRISPR/Cas9‐dependent genome editing to generate *let‐60(re378[*T35S*])*, which encodes T35S mutant LET‐60/Ras. LET‐60(T35S) mutants were superficially wild type in every regard except for increased stature (Figure 6D; S6J), similar to the AGE‐1(RBD). Unfortunately, we cannot generate the G12V T35S constitutively active LET‐60/Ras because G12V confers LIN‐45/Raf‐dependent lethality (J. Mardick and D. Reiner, unpublished). We propose that a LET‐60/Ras>AGE‐1/PI3Kcat imposes restriction of animal stature, such that reduced signal enables increased animal size and increased signal might decrease animal size.

### 2.7 Mutations in the Binding Interface of LET‐60/Ras>AGE‐1/PI3Kcat Reveal an Unexpected Role in the Dauer Diapause Decision

**FIGURE 7:**
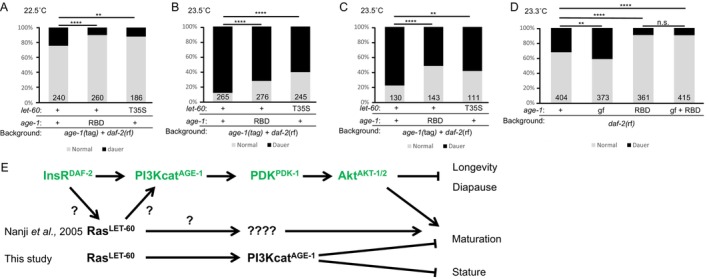
Putative defective association of LET‐60/Ras with the AGE‐1 RBD reduces dauer formation. (A–C) Percent dauer formation of *daf‐2(m577*rf*)* animals at different temperatures with *age‐1(tag)* alone, RBD mutation or *let‐60(T35S)* putative PI3K defective mutation. (The weaker *daf‐2(m577)* was used because *daf‐2(e1370*) conferred 100% dauer formation at these temperatures.) A was intentionally set lower while B and C are experimental replicates to account for incubator fluctuations. Animals in the same graph were scored concurrently. (D) The same experiment with untagged AGE‐1 reveals the same interactions. *p* values were calculated by Pearson's chi‐squared test. For *p* values, *< 0.05, **< 0.01, ***< 0.001, **** 0.0001. (E) A schematic of the dauer regulatory network, determined by a combination of published results and the novel interactions shown in this study. The core IIS pathway, DAF‐2/InsR>AGE‐1/PI3Kcat>PDK‐1/PDK>AKT‐1/2/Akt, is as found in *C. elegans* and other systems. Upstream sensory inputs converge on IIS. The pathway inhibits dauer diapause and promotes developmental maturation and reproduction. Prior experiments (Nanji et al. 2005) with G13E constitutively activating LET‐60/Ras suppressed dauer formation caused by reduction‐of‐function DAF‐2/InsR, arguing that LET‐60/Ras promotes reproductive fate at the expense of dauer diapause. In contrast, our results show that disruption of the LET‐60/Ras>AGE‐1/PI3K interaction reduces dauer formation in *daf‐2*(rf), indicating that this specific signaling input can promote dauer entry under these conditions. These findings are consistent with LET‐60>AGE‐1/PI3K signaling being context‐dependent and functionally separable from other LET‐60/Ras effector pathways, which may differentially regulate developmental outcomes depending on tissue, developmental stage, or effector engagement.

We used the T35S mutation in LET‐60/Ras and the AGE‐1(RBD) mutations in the RBD of AGE‐1/PI3Kcat to evaluate the role of LET‐60/Raf>AGE‐1/PI3Kcat signaling in dauer formation. Unexpectedly, we observed that under multiple temperatures and repeated assays, both T35S and AGE‐1(RBD) mutants reduced dauer formation (Figure 7A–C). This includes the AGE‐1(RBD) mutation in the untagged AGE‐1 (Figure 7D). Consequently, we propose that a LET‐60>AGE‐1 signaling input can promote dauer formation under these conditions, indicating that LET‐60→AGE‐1 signaling is functionally distinct from canonical IIS outputs. This distinction suggests that multiple inputs into AGE‐1/PI3K signaling have separable and context‐dependent effects on developmental decisions.

IIS components in *C. elegans* were identified largely through roles in control of the dauer diapause decision, resulting in a model of a DAF‐2/InsR>AGE‐1/PI3Kcat>PDK‐1/PDK>AKT‐1/2 cascade repressing dauer development and promoting reproductive development (figure 7D; Fielenbach and Antebi 2008). One study showed that the *let‐60(n1046*gf*)* G13E moderately activating mutation suppressed reduced function mutations in DAF‐2/InsR that precociously promote dauer formation (dauer constitutive, or Daf‐c; Nanji et al. 2005). Moreover, reduction of LET‐60/Ras function was predicted to weakly promote formation of dauers. Together, these results led to the model that LET‐60/Ras signaling functioned downstream of DAF‐2/InsR to repress dauer formation and promote reproductive development. This signal was speculated to be through AGE‐1/PI3Kact but that hypothesis could not be tested.

Our results contradict that finding. Predicted reduction of RBD function and reduction of LET‐60/Ras activation of AGE‐1/PI3Kcat binding of LET‐60/Ras both reduce dauer formation. Consequently, we hypothesize that LET‐60>AGE‐1 inhibit developmental progression to maturation at the expense of dauer formation, that is promote dauer diapause (Figure 7E).

A major methodological difference is that the previous mutations either reduced or increased all signaling functions of LET‐60/Ras. Perhaps these genetic perturbations alter multiple functions of LET‐60, at least one of which strongly promotes reproductive development, whereas our missense mutations more selectively alter LET‐60 and AGE‐1 functions. Alternatively, perhaps LET‐60 acts at multiple positions in the dauer regulatory network, or in multiple tissues in the animal. Some of these could be positive and some negative signals, and some via AGE‐1/PI3Kcat and others through another effector, like LIN‐45/Raf. Regardless, these findings illustrate the utility of using selective mutations driven by structural knowledge when interrogating biological functions in vivo.

### 2.8 Intramolecular Epistasis Using Gf and RBD Mutations in *Cis* Is Consistent With Gf Activities Requiring LET‐60 Binding

Both with and without the AGE‐1 C‐terminal tag, we find that introducing the RBD mutations into a gf mutant AGE‐1 blocks the effect of the gf mutation in ectopic 1° VPC induction (Figure 6E,F). We observed the same phenomenon with gf and RBD in untagged AGE‐1 in the *daf‐2(*rf*)*‐induced dauer formation assay: the AGE‐1(RBD) blocks the decreased dauer formation conferred by the gf mutation, indicating that the increased pathway output conferred by the E630K mutation depends on the intact LET‐60:AGE‐1 interaction (Figure 7D). These observations are that LET‐60 binding to the AGE‐1 RBD is required for the full activity of the E630K AGE‐1 allele in vivo.

## 3 Discussion

The goal of this study was to use structural insights to guide in vivo editing of the sole Class IA PI3K catalytic subunit in *C. elegans* , AGE‐1, and to define organismal consequences of discrete mutations. By combining structural threading with CRISPR/Cas9‐dependent genome editing, we introduced a functional C‐terminal fluorescent tag, an activating mutation modeled on oncogenic PI3Kcat/p110α alleles, and a Ras‐binding–deficient mutation. These tools allowed us to probe AGE‐1 function in lifespan, vulval development, growth, and dauer entry.

Our tagging strategy highlights the value of structure‐based design for engineering large multidomain proteins. By introducing an extended flexible linker between AGE‐1 and the fluorescent protein, we avoided steric clashes predicted from crystallographic studies and supported by AlphaFold3 models. The tagged protein retained full function in lifespan and vulval assays, including in sensitized *let‐60(n1046*gf*)* and *daf‐2(*rf*)* genetic backgrounds, allowing accurate visualization of endogenous expression. This tagged protein now provides a versatile platform for future structure–function studies and biochemical analyses. Likewise, structural modeling of the helical and Ras‐binding domains enabled us to design and generate alleles that either phenocopied known oncogenic mutations or selectively disrupted Ras interaction without destabilizing the protein. These results underscore the power of structural information to produce precise, interpretable alleles for whole‐animal studies.

### 3.1 Mechanisms of AGE‐1 Activation

Our findings that the RBD mutation blocks the effect of the gf mutation in AGE‐1 for excess 1° induction and dauer formation phenotypes is consistent with a requirement of RBD binding by LET‐60 for phenotypes induced by likely constitutively active AGE‐1. We propose that while AGE‐1 is active, it needs to be recruited to the PM by activated LET‐60 to encounter PIP_2_ in the PM to convert to PIP_3_ and propagate signal. A *caveat* to this model is that AGE‐1(RBD) does not alter excess 1° induction by *let‐60(n1046*gf*)*. Perhaps LET‐60>AGE‐1/PI3K signaling is context‐dependent: it is not required for VPC fate modulation under these assay conditions but is functionally significant in other physiological outputs, including regulation of organismal growth and dauer entry.

### 3.2 Animal Stature

An unexpected finding was the increase in body size in animals carrying AGE‐1(RBD) substitutions that reduce LET‐60:AGE‐1 interaction, as well as in animals carrying the complementary LET‐60(T35S) effector‐binding mutation. In mammalian systems, PI3K signaling is generally associated with promotion of cellular growth; however, our results support the model that, in *C. elegans* , a LET‐60>AGE‐1/PI3K signaling input can function to constrain organismal growth. These conclusions are based on interface‐directed substitutions that preserve AGE‐1 stability and expression, arguing against general loss‐of‐function effects. The magnitude and consistency of the size phenotype across independent perturbations of the LET‐60:AGE‐1 interface support a specific role for this interaction in regulating growth.

In other systems, including *Drosophila*, insulin receptor signaling through PI3K and mTORC1 is a major positive regulator of cell and body size (Oldham and Hafen 2003). In *C. elegans* , nutritional input and IIS similarly influence animal size (McCulloch and Gems 2003; So et al. 2011). Within this broader framework, our results point to a specific LET‐60>AGE‐1 signaling input that opposes growth‐promoting IIS outputs, consistent with context‐dependent integration of signaling pathways rather than a uniform effect of PI3K activation on size.

Our dauer assays similarly implicate complexity in Ras–PI3K pathway integration. Prior models place LET‐60 downstream of DAF‐2/InsR in promoting reproductive development and suppressing dauer entry (Nanji et al. 2005). In contrast, perturbation of the LET‐60:AGE‐1 interface reduced dauer formation, consistent with this signaling input promoting dauer entry under certain conditions. These observations are most parsimoniously explained by multiple, context‐dependent AGE‐1 inputs, potentially differing across tissues or developmental stages. Dissecting these contributions will require spatially and temporally resolved genetic approaches.

In summary, our structure‐guided engineering of AGE‐1 demonstrates how defined, interface‐level mutations can be used to dissect domain‐specific functions in vivo. The E630K substitution increases AGE‐1 pathway output, as assessed by lifespan, DAF‐16::GFP localization, and VPC induction. In contrast, targeted perturbation of the LET‐60:AGE‐1 interface reduces physical interaction in vivo and reveals a specific role for this interaction in regulating organismal growth and dauer entry without broadly impairing AGE‐1 function. Together, these findings illustrate how integration of structural modeling with endogenous genome editing enables precise functional dissection of conserved signaling proteins in a physiological context.

## 4 Methods

### 4.1 *C. elegans* Handling and Genetics

Strains used in this study were derived from the N2 Bristol wild type and grown at 20°C on NGM plates seeded with *E. coli* strain OP50. *C. elegans* strains used are listed in Table S1, Oligonucleotides in Table S2, CRISPR reagents in Table S3. Nomenclature is as described (Tuli et al. 2018).

### 4.2 CRISPR/Cas9‐Dependent Genome Editing

Guide RNAs were selected using a combination of three criteria. First, we prioritized sequence features shown to improve Cas9 activity: where possible, guanine (G) rather than thymine (T) nucleotides were chosen at positions −1/−2/−4, or the GCGG sequence in preference over Ts at positions −1 through −4 (Farboud and Meyer 2015; Wang et al. 2014). Second, predicted specificity and efficiency were evaluated using the CRISPOR algorithm (https://crispor.gi.ucsc.edu/; Concordet and Haeussler 2018), which incorporates the original MIT specificity score. Third, predicted efficiency was further assessed using the WU‐CRISPR algorithm (http://crisprdb.org/wu‐crispr/; Wong et al. 2015).

Injection mixes were formulated as described (Fakieh and Reiner 2025; Ghanta et al. 2021). Specifically, we used final concentrations of 0.25 mg/mL SpCas9 (PNABio), 0.1 mg/mL universal tracrRNA, 0.028 mg/mL crRNAs for the *dpy‐10* co‐CRISPR marker and the edit of interest, repair templates for the dominant *dpy‐10(cn64*gf*)* co‐CRISPR marker and the repair template of interest to 3.3 mM (Table S3). Mixes were assembled in an RNAse‐free bench space and incubated at 37°C for 15 min.

F1 animals expressing the *dpy‐10(cn64*gf*)* Rol co‐CRISPR marker (Arribere et al. 2014) were picked singly to plates. After F2 embryos were laid, F1s were picked into tubes—sometimes singly and sometimes in pools of 2—and lysed for single‐worm PCR. Non‐Rol or ‐Dpy F2 animals from PCR positives were PCR amplified for homozygous edits. The edited region from resulting homozygotes was amplified and Sanger sequenced.

Sequences encoding an N‐terminal extension were inserted at the 5′ end of the endogenous *let‐60* gene in two steps. First, we inserted sequences containing the sgRNA for introducing the *dp‐10(cn64*gf*)* co‐CRISPR marker (Arribere et al. 2014). This reagent has been published (Gibney et al. 2026). Second, into this efficient site we used the corresponding sgRNA introduced sequences encoding 3xFLAG (Figure S6D). For editing *age‐1*, the CRISPR/Cas9‐dependent insertion was at the C‐term, with a 30‐residue linker consisting of 10xGly‐Ala‐Ser, followed by mNeonGreen (mNG) and a 2xHA epitope tag (Figure 2A).

### 4.3 Imaging

Live animals were mounted on 3% agar pads with 5 μL of 2 mg/mL tetramisole in M9 buffer. Differential Interference Contrast (DIC) images were captured using the Nikon 224 Eclipse Ni Microscope with a 60× oil objective. Confocal fluorescent micrographs were captured using a Ti2‐Nikon inverted microscope equipped with a Yokogawa CSU‐W1 Spinning Disk and a Photometrics Prime BSI camera. Green fluorescence (mNeonGreen) was captured at 488 nm. NIS Elements Version 4.30 software was used to capture and process images.

### 4.4 Quantifying Animal Phenotypes

Synchronized embryos were obtained by picking adults (24 h post‐late L4) onto plates with food and picking off 1 h later.

Animal size was quantified using synchronized adults 72 h after egg laying. DIC photos were taken using a spinning disk confocal microscope with the 40× objective. Length of animals was analyzed using NIS Elements Version 4.30 software. Length was measured as distance from the anterior to posterior (A‐P) end and width as the ventral to dorsal (V‐D) at the A‐P midpoint where the vulva is located.

For lifespan assays, 20 young adult animals (24 h after mid L4) were picked to each plate, multiple plates for each genotype. Day 0 was thus mid L4. Animals were transferred and assessed every 24 h after initial picking until production of embryos ceased. Total number of surviving animals was quantified every other day. Animals that could not be scored for lifespan, for example they swam up the sides of the plate or under the agar, were censored from the aging data (Sutphin and Kaeberlein 2009).

Strains for dauer assays were propagated at 15°C. Ten young but gravid adults were picked to each plate for egg laying at the temperature indicated and picked off for approximate synchrony of embryos. Dauer versus adult animals for each plate were quantified 72 h later.

The role of AGE‐1/PI3Kcat signaling in patterning of cell fate of vulval precursor cells (VPCs) was scored as described (Fakieh and Reiner 2025; Shin et al. 2018). Briefly, we counted ectopic 1° VPCs induced by gain of function of the *C. elegans* ortholog of Ras, *let‐60(n1046*gf*)*, with and without edits in *age‐1*. Late L4 animals were mounted and imaged via DIC (see above). Ectopic 1°s were identified as single‐lobed invaginations compared to the three‐lobed invagination of the normal vulva. In strains harboring *let‐60(n1046*gf*)*, we have observed drift of the strength of the phenotype of ectopic 1° pseudovulvae, typically becoming more severe. As previously described, we carefully avoided multi‐generational plate culture of animals, either analyzing freshly from a thawed strain, recently isolated as a homozygote from a cross, or recovered from a starved, parafilmed plate that was stored immediately after thawing. Strains not fitting the established baseline range of 1.2–1.5 ectopic 1° pseudovulvae are discarded and the strains restarted as described (Zand et al. 2011).

For the DAF‐16::GFP (*zIs356[daf‐16p>daf‐16a/b::GFP+rol‐6(su1006d)]*) nuclear localization assay, L1 animals were mounted and imaged via spinning disc confocal microscopy with the 60× oil lens at 488 nm (5 μL of 0.04 mg/mL tetramisole in M9 buffer was used as anesthetic to avoid DAF‐16::GFP stimulation observed at higher concentrations). NIS Elements Version 4.30 software was used to capture and process images. For heat shock experiments, synchronized L1 animals were put in a 32°C incubator for 30 min before experiments. For both assays, photomicrographs were scored blind based on the rubric in the figure legend.

### 4.5 Detection of Tagged Endogenous Protein

For immunoblotting, mixed stage animals were washed from plates using M9 buffer and lysed in 4% SDS loading buffer by boiling at 92°C for 5 min. Lysates were run on 4%–15% SDS gel (Bio‐Rad) and blotted on Immobilon‐P Membrane, PVDF (EMD Millipore, IPVH00010). Anti‐HA antibody (Proteintech 51064‐2‐AP), anti‐FLAG antibody (Sigma‐Aldrich F1804), anti‐Ras antibody (cell signaling #67648) and anti‐α‐tubulin antibody (Sigma‐Aldrich T6199) were diluted 1:3000 in blocking buffer (6% w/v non‐fat dry milk in PBST). HRP‐conjugated goat anti‐mouse secondary antibody (MilliporeSigma 12‐349) and HRP HRP‐conjugated goat anti‐Rabbit secondary antibody (Cell signaling #7074) were diluted in 1:5000 in blocking buffer. Chemiluminescent detection was performed using ECL reaction (Thermo Fisher Scientific), and detected using the Bio‐Rad Chemidoc MP Imaging System Hood III. For co‐immunoprecipitation, animals were lysed using a sonicator (QSONICA Q500) with 25% Amplitude at 10 s, followed by 60 s cool down and repeated six times. Lysate was incubated with anti‐FLAG antibody (Cell Signaling #14793) and Dynabeads protein A (Invitrogen #10002D) in 4°C overnight and detected by western blot as described. Washing buffer consisted of 60 mM HEPES (pH 7.4), 150 mM KCl, 0.2% Triton, 4 mM MgCl_2_, 10% glycerol and 2 mM DTT.

### 4.6 Protein Structural Analysis and Model Construction

Structure threading used PHYRE2.2 (Powell et al. 2025) to obtain a model of the AGE‐1+AAP‐1 structure, which was then used to 3D align with earlier PI3KCA/p85 structures. The sequence of LET‐60 was threaded onto the structure of MRAS bound to PI3KCA (Czyzyk et al. 2025). Structures were visualized with PyMol 2.6 (Schrödinger LLC) and earlier versions (https://www.pymol.org/). Protein Data Bank accession numbers used were 4OVU, 4OVV, 9B4T (Burley et al. 2025).

### 4.7 Software and Statistical/Data Analysis

Data analysis and graphing were performed using Prism 9 (version 9.0.1) and Microsoft Excel (version 16.45). Aging assay data analysis and the Kaplan–Meier graph used the online survival analyzing tool on https://sbi.postech.ac.kr/oasis2/ (Han et al. 2016). Protein alignments used Clustal Omega; domain analysis used SMART and Prosite.

## Author Contributions

Conception, design, acquisition of data, analysis and interpretation of data: Y.W., D.J.R.; drafting and revising the manuscript: Y.W., D.J.R.; critical technical mentoring and/or generation of key reagents: T.D., N.R.R.; structural analysis: K.L.R., Y.W., D.J.R.

## Funding

This work was supported by the National Institute of General Medical Sciences (Grant R35GM144237) and National Cancer Institute (Grant R03CA289854).

## Ethics Statement

All ethics and safety guidelines were observed.

## Conflicts of Interest

The authors declare no conflicts of interest.

## Supporting information


**Figure S1:** Structural model of human HRAS, PI 3‐Kinase catalytic alpha, and PIK3R1 p85. (A) A *pre hoc* model of *C. elegans* AGE‐1+AAP‐1 threaded onto PI3Kcat alpha and p85 with an HRAS structure superimposed. HRAS is bound to nonhydrolyzable GTP analog GMPPNP, is colored in pale green, and the Switch II region that binds effectors and shifts upon GTP binding is colored in red. This model of threaded AGE‐1+AAP‐1 bound to HRAS specifically led us to tag the endogenous AGE‐1 protein at the C‐terminus. The plasma membrane in this model is located above the enzyme, as indicated by the C‐terminus (indicated) of HRAS, which is directed to the PM. The C‐terminus is predicted to be sufficiently far from the PM to avoid steric interference of an ~27 kDa fluorescent protein, with a 30 residue GASx10 linker sequence added to decrease risk of interference. The N‐terminal SH2 domain (nSH2) is shown partially structured. The C‐terminal SH2 domain (cSH2) is not shown, as is typical, as are the domains of p85 missing in p50 and p55. (B) A 180° rotation of the structural model reveals the predicted location of the N‐terminus. Tagging with an FP might interfere with general PM association. (C) The unstructured N‐terminal extensions of *C. elegans* AGE‐1 and Drosophila PI3K92E are shown, with Arginine residues bolded and in green. We hypothesize that the basic N‐terminus of AGE‐1 provides an electrostatic charge with the acidic PM, which might be destabilized by an FP tag, another reason to avoid the N‐terminus of AGE‐1 for tagging.
**Figure S2:** Spinning disk confocal photomicrographs (488 nm) of *age‐1(re353[age‐1::linker::mNG::2xHA])* animals. (A) Adult. (B) Late fourth larval stage (L4). White arrow = nerve ring neuropil (a bundle of many neurites) and perhaps the excretory duct/pore. White arrow = nerve ring neuropil (a bundle of many neurites.) Yellow arrowhead = morphogenetic vulva. (G) Third larval stage. White arrowhead = P6.p of the VPCs, flanked by other VPCs along the ventral surface. See Figure 2: bright green spots are autofluorescence from intestinal lipid droplets. Dark circles are nuclei. (H) Embryos. We consistently observe speckles in embryos, presumably cytosolic, but do not know the source. White scale bars = 100 μm. Green scale bar = 50 μm.
**Figure S3:** Support for the E630K mutation in AGE‐1. (A) Schematic of the edited age‐1 gene and protein with tag (see Figure 2A) or with the E630K mutation. (B) An alignment of the entire PIK/helical domains. Hs = *Homo sapiens* , Dm = *Drosophila melanogaster* , Ce = *Caenorhabditis elegans* . Human E542 and E545 residues altered to K in oncogenic lesions in the PIK/helical domain are shown in red, the nematode E630 residue altered to K in the gain‐of‐function is shown in blue. Each protein has four acidic residues in this region, just clustered in nematodes and more interspersed in humans. While the helices in the predicted structure are of the PIK/helical domain reasonably well conserved, the primary sequence is not. (C) Immunoblotting with anti‐HA indicates the E630K mutation did not alter protein stability of expression. The western shown is the same shown for sizing in Figure 2B, from which the E630K lane and the RBD lanes (see Figure S5C) were cropped. (D) Kaplan–Meier curves show percent survival of wild‐type versus *age‐1(re489*gf) “tagless” animals, analyzed concurrently. *N* = 120 (43 censored) for wild type and *N* = 120 (30 censored) for *age‐1(gf*). (E, F) spinning disk confocal photomicrograph (488 nm) of an optical section of an *age‐1(re*353[tag+gf]*)* adult (E) and L4 animal (F). ***p < 0.001. Scale bars are 100 μm.
**Figure S4:** (A) *age‐1*(gf), without tag, significantly decreased nuclear localization of DAF‐16::GFP after 30 min heat shock at 32°C. ***≤ 0.001, calculated by Mann–Whitney test.
**Figure S5:** (A) Raw data for Figure 5F.
**Figure S6:** Support for the RBD in AGE‐1/PI3Kcat. (A) Schematic of the edited *age‐1* gene and protein with tag (see Figure 2A, S3A) or with the R303E K304E mutation. (B) An alignment of entire RBD domains. Hs = *Homo sapiens* , Dm = *Drosophila melanogaster*, Ce = *Caenorhabditis elegans* . Human T208 and K227 residues altered to D and A, respectively, in the human RBD deficient mutant (Gupta et al. 2007) are shown in pink. *Drosophila* T231, K250, R253, and K257 altered to D, A, A, and A, respectively in the *Drosophila* RBD deficient mutant (Orme et al. 2006) are shown in green. With the relatively poor conservation of the RBD, we did not mutate these specifically but rather mutated structure‐based residues. The nematode R303 K304 residues altered to E in the RBD reduction‐of‐function mutant are shown in blue. (C) Immunoblotting with ⍺‐HA indicates the R303E K304E mutations did not alter protein stability orlink expression. The western shown is the same shown for sizing in Figure 2B, where the E630K lane and the RBD lanes were cropped. (D) Schematic of construction of *3xFLAG::d10::let‐60* double sequential tag via *d10* (the sequences of the guide RNAi in *dpy‐10* used to edit in the *cn64*gf Rol co‐CRISPR marker) followed by the insertion of sequences encoding 3xFLAG into the center of *d10* (Gibney et al. 2026). (E, F) AGE‐1::linker::mNG::2xHA(RBD) is expressed at the same levels in adults (E) and L4 larvae (F). (G) Validation of d10 and d10::3xFLAG insertions at the N‐terminus of endogenous LET‐60 using a monoclonal antibody raised against a human H/M/RRAS epitope (see Methods). (H) Western blot of bead‐bound FLAG::LET‐60 co‐immunoprecipitating AGE‐1::HA, with single tag controls and input controls. (I) Animal width in microns as measured from DIC photomicrographs for RBD and gf mutations in AGE‐1 as shown in Figure 6C. (J) Animal width in microns as measured from DIC photomicrographs for the LET‐60 T35S putative Raf‐selective mutant vs. wild type as shown in Figure 6D. (K) Kaplan–Meier curve for lifespan of wild type vs. untagged AGE‐1(RBD). *N* = 140 (43 censored), animal = 43 for wild type, *N* = 120 (24 censored) for *age‐1* RBD. (L) Kaplan–Meier curve for lifespan of *age‐1*(tag) versus, *age‐1*(tag+RBD) mutants in the *daf‐2*(*e1370*rf) background. *N* = 160 (43 censored) for *age‐1* tag + *daf‐2*(*e1370*rf) and *N* = 120 (36 censored) for *age‐1* tag+RBD + *daf‐2*(*e1370*rf). *p* value for animal length and width was calculated using *t‐*test, and animal aging was calculated using Log‐rank (Mantel‐Cox) test and ANOVA. n.s. = not significant, **≤ 0.01, ****≤ 0.0001.

**Table S1:** Strain list.
**Table S2:** Primer list.
**Table S3:** CRISPR reagents.

## Data Availability

All data are presented in the manuscript; no large data sets requiring upload were generated. All reagents are available upon request or are being sent to key repositories, for example CGC, Addgene.org.
