# Supplementary material for: Aging, Dauer, and Stature Phenotypes Are Conferred by Structure‐Directed Missense Mutations in the Endogenous AGE‐1/Phosphatidylinositol 3‐Kinase Catalytic Subunit
Source: Aging Cell. 2026 Jun 18;25(6):e70571. doi: 10.1111/acel.70571 (PMC13277760; doi:10.1111/acel.70571)
Supplement: Supplementary file 1 — Figure S1: Structural model of human HRAS, PI 3‐Kinase catalytic alpha, and PIK3R1 p85. (A) A pre hoc model of C. elegans AGE‐1+AAP‐1 threaded onto PI3Kcat alpha and p85 with an HRAS structure superimposed. HRAS is bound to nonhydrolyzable GTP analog GMPPNP, is colored in pale green, and the Switch II region that binds effectors and shifts upon GTP binding is colored in red. This model of threaded AGE‐1+AAP‐1 bound to HRAS specifically led us to tag the endogenous AGE‐1 protein at the C‐terminus. The plasma membrane in this model is located above the enzyme, as indicated by the C‐terminus (indicated) of HRAS, which is directed to the PM. The C‐terminus is predicted to be sufficiently far from the PM to avoid steric interference of an ~27 kDa fluorescent protein, with a 30 residue GASx10 linker sequence added to decrease risk of interference. The N‐terminal SH2 domain (nSH2) is shown partially structured. The C‐terminal SH2 domain (cSH2) is not shown, as is typical, as are the domains of p85 missing in p50 and p55. (B) A 180° rotation of the structural model reveals the predicted location of the N‐terminus. Tagging with an FP might interfere with general PM association. (C) The unstructured N‐terminal extensions of C. elegans AGE‐1 and Drosophila PI3K92E are shown, with Arginine residues bolded and in green. We hypothesize that the basic N‐terminus of AGE‐1 provides an electrostatic charge with the acidic PM, which might be destabilized by an FP tag, another reason to avoid the N‐terminus of AGE‐1 for tagging. Figure S2: Spinning disk confocal photomicrographs (488 nm) of age‐1(re353[age‐1::linker::mNG::2xHA]) animals. (A) Adult. (B) Late fourth larval stage (L4). White arrow = nerve ring neuropil (a bundle of many neurites) and perhaps the excretory duct/pore. White arrow = nerve ring neuropil (a bundle of many neurites.) Yellow arrowhead = morphogenetic vulva. (G) Third larval stage. White arrowhead = P6.p of the VPCs, flanked by other VPCs along the ventral surfac [file ACEL-25-e70571-s001.pptx]

## Slide 1
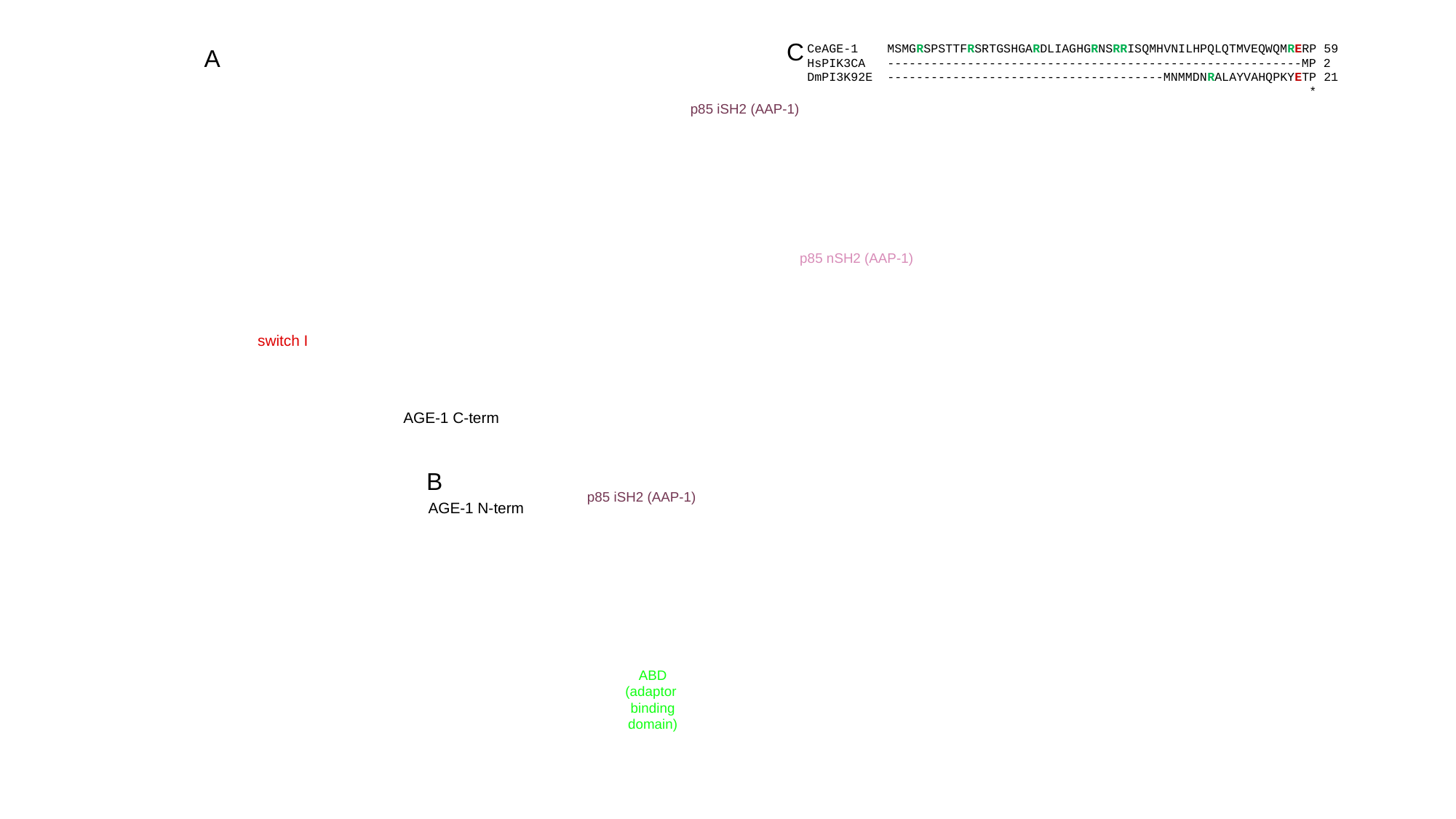

A
p85 iSH2 (AAP-1)
p85 nSH2 (AAP-1)
B
p85 iSH2 (AAP-1)
ABD
(adaptor
binding
domain)
C
CeAGE-1 MSMGRSPSTTFRSRTGSHGARDLIAGHGRNSRRISQMHVNILHPQLQTMVEQWQMRERP 59
HsPIK3CA ---------------------------------------------------------MP 2
DmPI3K92E --------------------------------------MNMMDNRALAYVAHQPKYETP 21
 *
switch I
AGE-1 C-term
AGE-1 N-term

## Slide 2
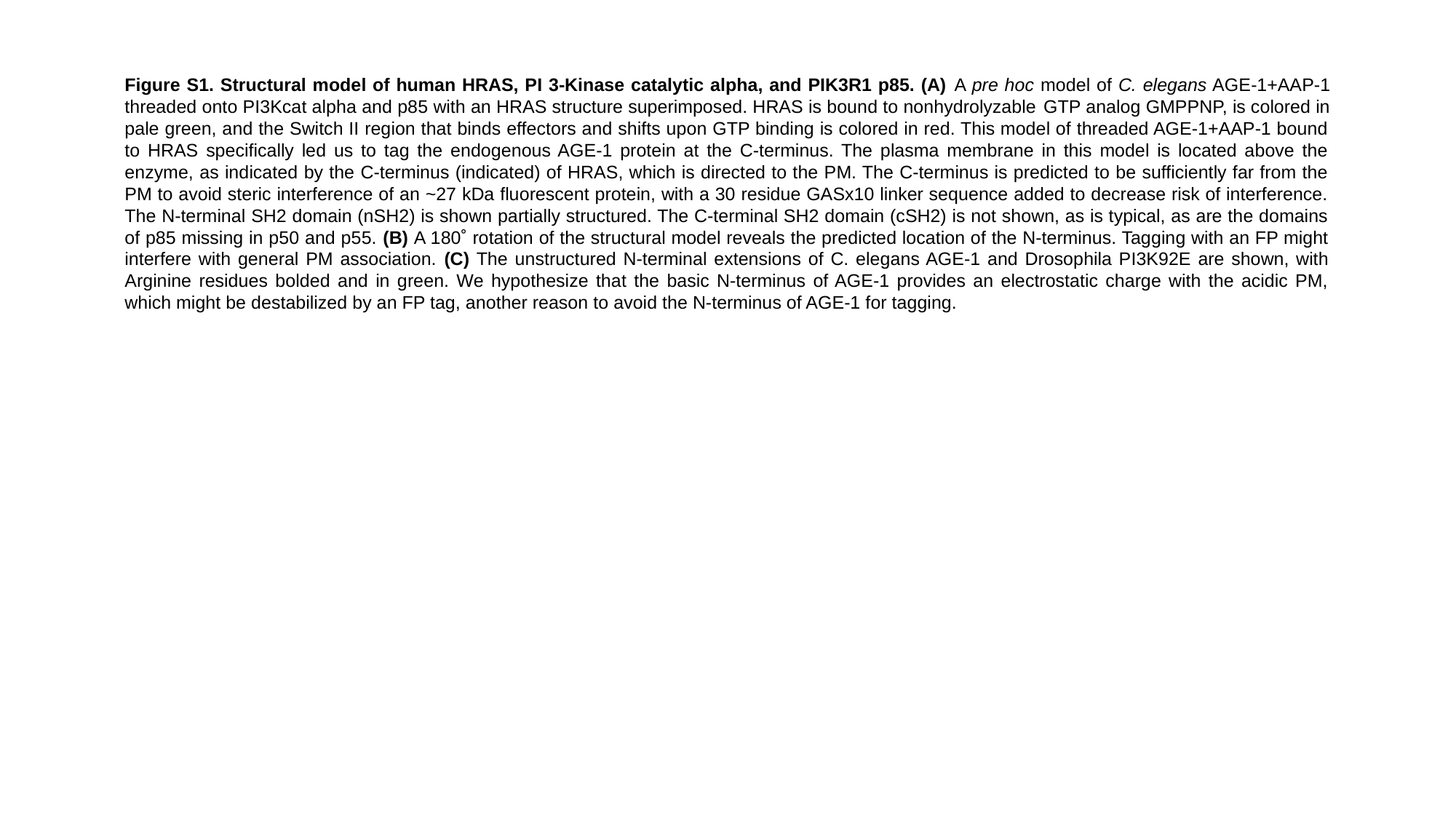

Figure S1. Structural model of human HRAS, PI 3-Kinase catalytic alpha, and PIK3R1 p85. (A) A pre hoc model of C. elegans AGE-1+AAP-1 threaded onto PI3Kcat alpha and p85 with an HRAS structure superimposed. HRAS is bound to nonhydrolyzable GTP analog GMPPNP, is colored in pale green, and the Switch II region that binds effectors and shifts upon GTP binding is colored in red. This model of threaded AGE-1+AAP-1 bound to HRAS specifically led us to tag the endogenous AGE-1 protein at the C-terminus. The plasma membrane in this model is located above the enzyme, as indicated by the C-terminus (indicated) of HRAS, which is directed to the PM. The C-terminus is predicted to be sufficiently far from the PM to avoid steric interference of an ~27 kDa fluorescent protein, with a 30 residue GASx10 linker sequence added to decrease risk of interference. The N-terminal SH2 domain (nSH2) is shown partially structured. The C-terminal SH2 domain (cSH2) is not shown, as is typical, as are the domains of p85 missing in p50 and p55. (B) A 180˚ rotation of the structural model reveals the predicted location of the N-terminus. Tagging with an FP might interfere with general PM association. (C) The unstructured N-terminal extensions of C. elegans AGE-1 and Drosophila PI3K92E are shown, with Arginine residues bolded and in green. We hypothesize that the basic N-terminus of AGE-1 provides an electrostatic charge with the acidic PM, which might be destabilized by an FP tag, another reason to avoid the N-terminus of AGE-1 for tagging.

## Slide 3
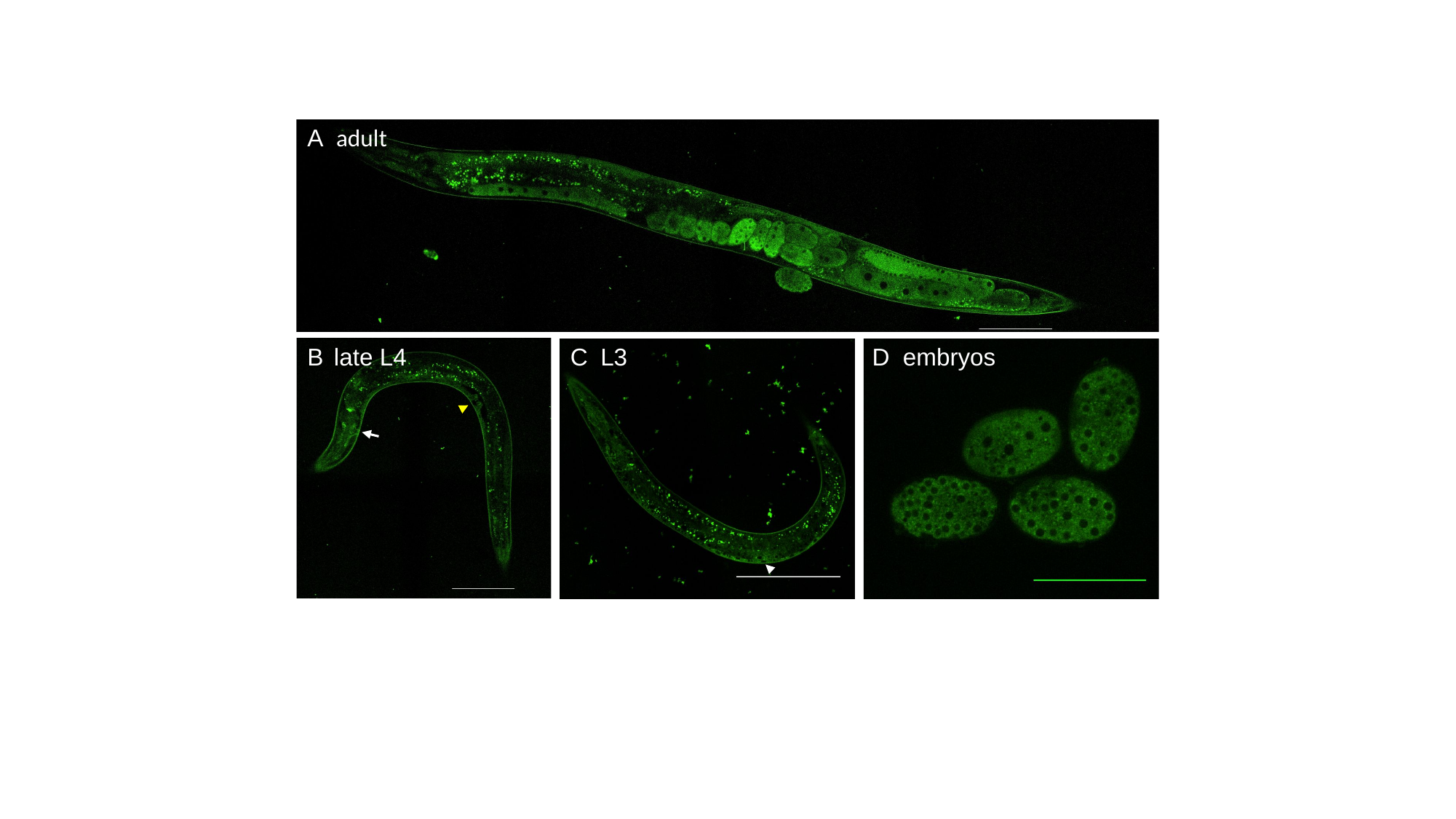

adult
A
H
B
late L4
C
L3
D
embryos

## Slide 4
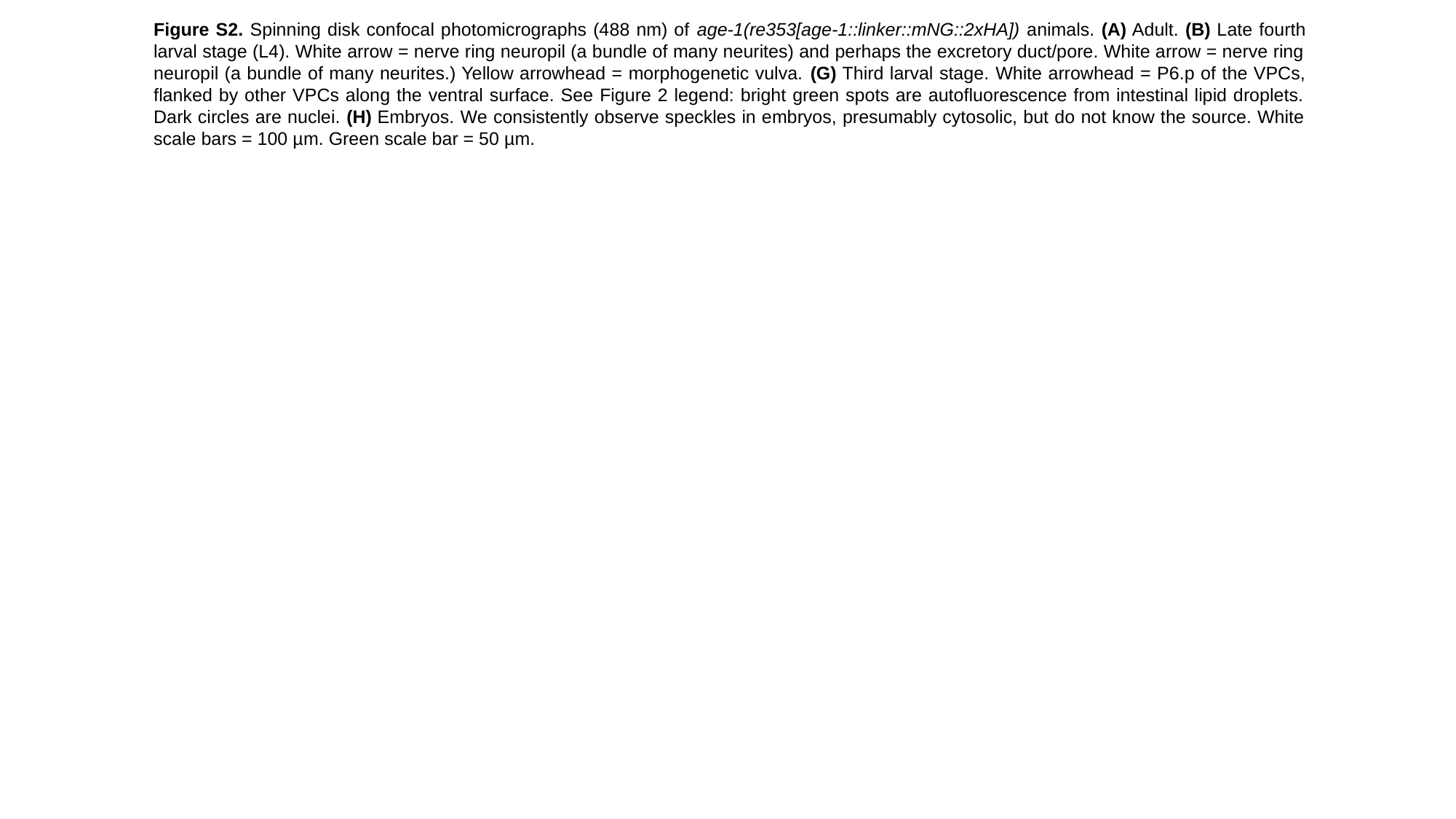

Figure S2. Spinning disk confocal photomicrographs (488 nm) of age-1(re353[age-1::linker::mNG::2xHA]) animals. (A) Adult. (B) Late fourth larval stage (L4). White arrow = nerve ring neuropil (a bundle of many neurites) and perhaps the excretory duct/pore. White arrow = nerve ring neuropil (a bundle of many neurites.) Yellow arrowhead = morphogenetic vulva. (G) Third larval stage. White arrowhead = P6.p of the VPCs, flanked by other VPCs along the ventral surface. See Figure 2 legend: bright green spots are autofluorescence from intestinal lipid droplets. Dark circles are nuclei. (H) Embryos. We consistently observe speckles in embryos, presumably cytosolic, but do not know the source. White scale bars = 100 µm. Green scale bar = 50 µm.

## Slide 5
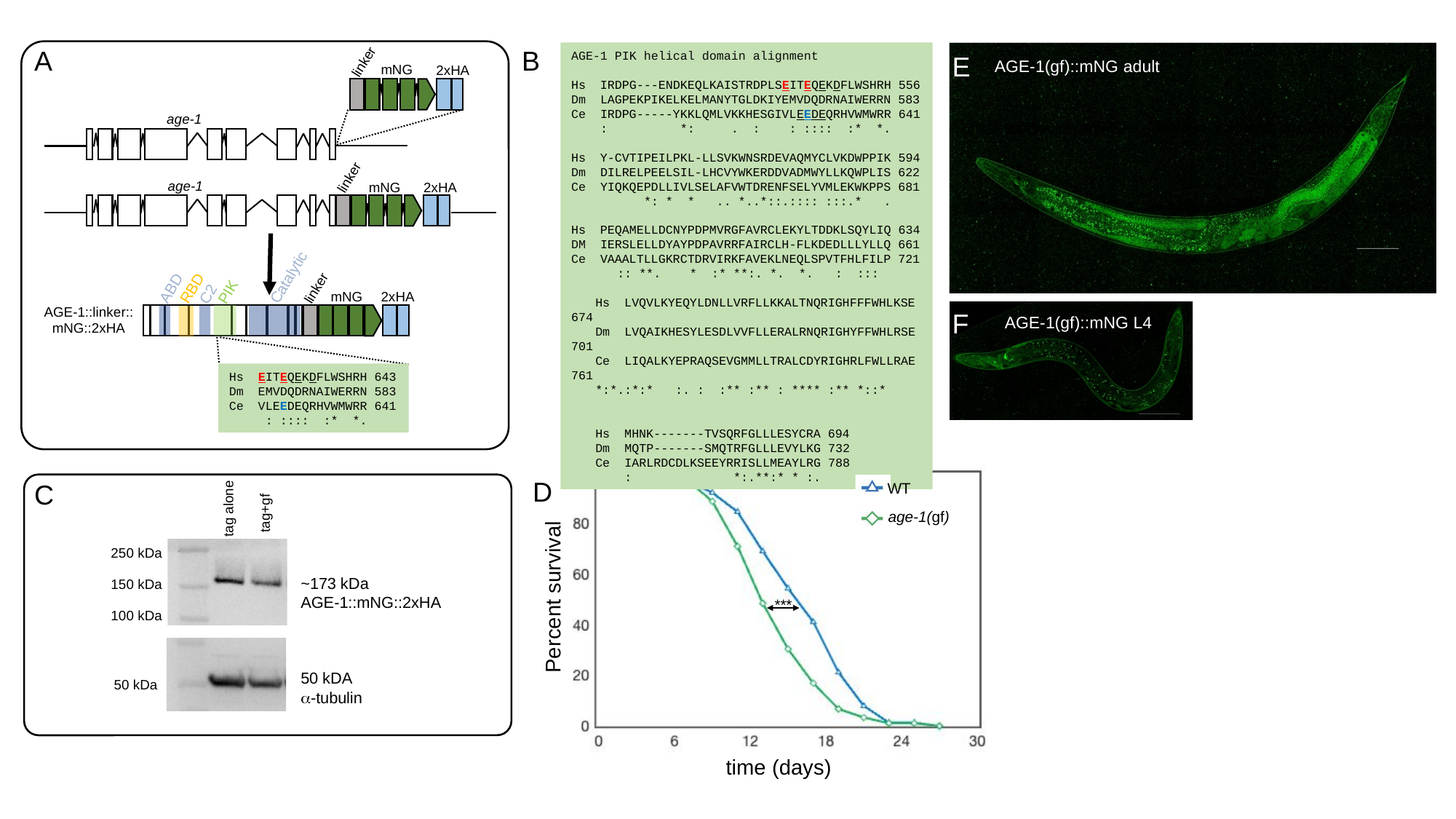

A
B
AGE-1 PIK helical domain alignment
Hs IRDPG---ENDKEQLKAISTRDPLSEITEQEKDFLWSHRH 556
Dm LAGPEKPIKELKELMANYTGLDKIYEMVDQDRNAIWERRN 583
Ce IRDPG-----YKKLQMLVKKHESGIVLEEDEQRHVWMWRR 641
  : *: . : : :::: :* *.
Hs Y-CVTIPEILPKL-LLSVKWNSRDEVAQMYCLVKDWPPIK 594
Dm DILRELPEELSIL-LHCVYWKERDDVADMWYLLKQWPLIS 622
Ce YIQKQEPDLLIVLSELAFVWTDRENFSELYVMLEKWKPPS 681
 *: * * .. *..*::.:::: :::.* .
Hs PEQAMELLDCNYPDPMVRGFAVRCLEKYLTDDKLSQYLIQ 634
DM IERSLELLDYAYPDPAVRRFAIRCLH-FLKDEDLLLYLLQ 661
Ce VAAALTLLGKRCTDRVIRKFAVEKLNEQLSPVTFHLFILP 721
 :: **. * :* **:. *. *. : :::
Hs LVQVLKYEQYLDNLLVRFLLKKALTNQRIGHFFFWHLKSE 674
Dm LVQAIKHESYLESDLVVFLLERALRNQRIGHYFFWHLRSE 701
Ce LIQALKYEPRAQSEVGMMLLTRALCDYRIGHRLFWLLRAE 761
*:*.:*:* :. : :** :** : **** :** *::*
Hs MHNK-------TVSQRFGLLLESYCRA 694
Dm MQTP-------SMQTRFGLLLEVYLKG 732
Ce IARLRDCDLKSEEYRRISLLMEAYLRG 788
 : *:.**:* * :.
E
linker
AGE-1(gf)::mNG adult
mNG
2xHA
age-1
linker
age-1
2xHA
mNG
PIK
Catalytic
ABD
RBD
C2
linker
2xHA
mNG
AGE-1::linker:: mNG::2xHA
F
AGE-1(gf)::mNG L4
Hs EITEQEKDFLWSHRH 643
Dm EMVDQDRNAIWERRN 583
Ce VLEEDEQRHVWMWRR 641
  : :::: :* *.
D
***
time (days)
Percent survival
C
WT
tag alone
tag+gf
age-1(gf)
250 kDa
~173 kDa
AGE-1::mNG::2xHA
150 kDa
100 kDa
50 kDA
a-tubulin
50 kDa

## Slide 6
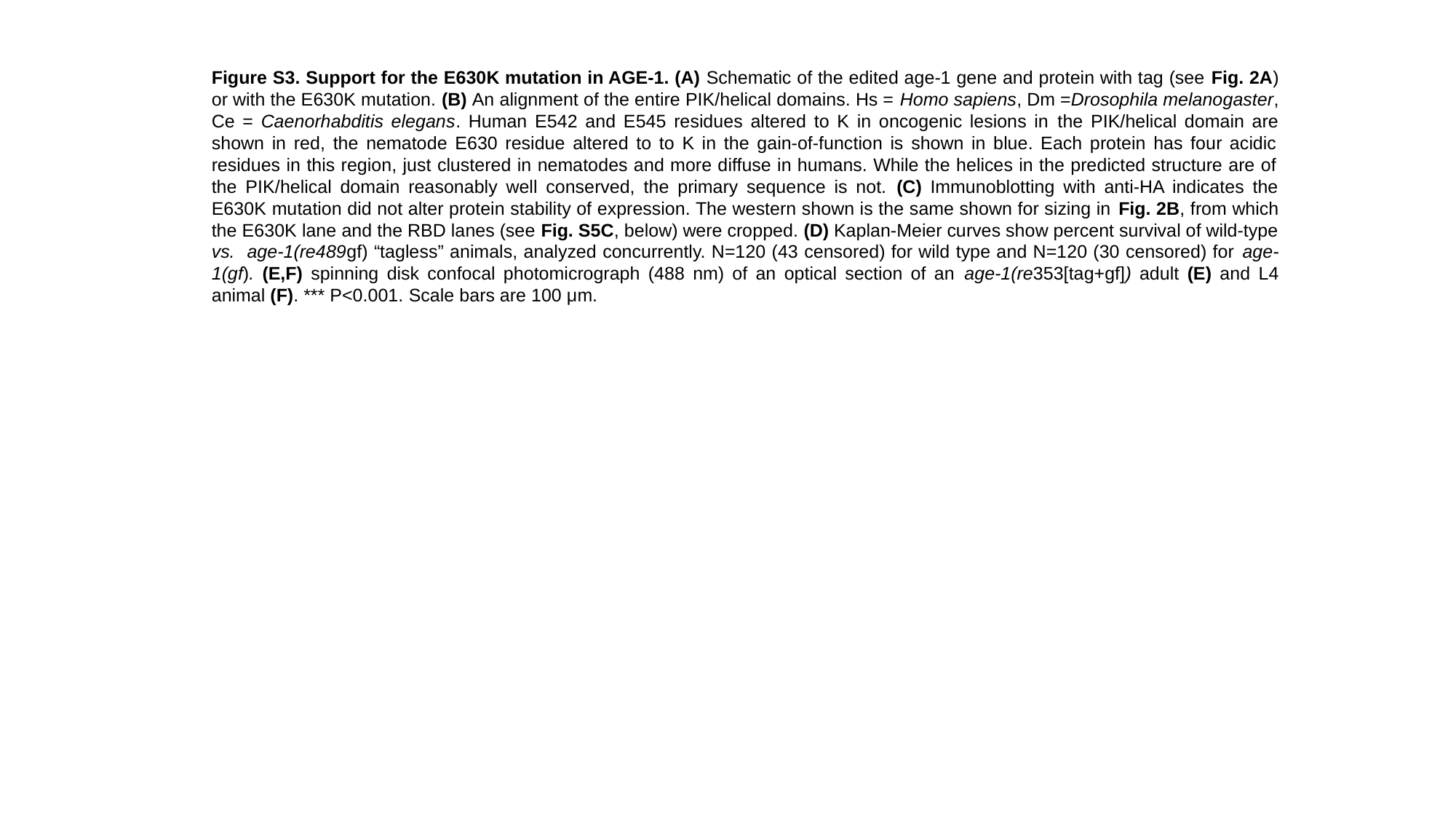

Figure S3. Support for the E630K mutation in AGE-1. (A) Schematic of the edited age-1 gene and protein with tag (see Fig. 2A) or with the E630K mutation. (B) An alignment of the entire PIK/helical domains. Hs = Homo sapiens, Dm =Drosophila melanogaster, Ce = Caenorhabditis elegans. Human E542 and E545 residues altered to K in oncogenic lesions in the PIK/helical domain are shown in red, the nematode E630 residue altered to to K in the gain-of-function is shown in blue. Each protein has four acidic residues in this region, just clustered in nematodes and more diffuse in humans. While the helices in the predicted structure are of the PIK/helical domain reasonably well conserved, the primary sequence is not. (C) Immunoblotting with anti-HA indicates the E630K mutation did not alter protein stability of expression. The western shown is the same shown for sizing in Fig. 2B, from which the E630K lane and the RBD lanes (see Fig. S5C, below) were cropped. (D) Kaplan-Meier curves show percent survival of wild-type vs. age-1(re489gf) “tagless” animals, analyzed concurrently. N=120 (43 censored) for wild type and N=120 (30 censored) for age-1(gf). (E,F) spinning disk confocal photomicrograph (488 nm) of an optical section of an age-1(re353[tag+gf]) adult (E) and L4 animal (F). *** P<0.001. Scale bars are 100 μm.

## Slide 7
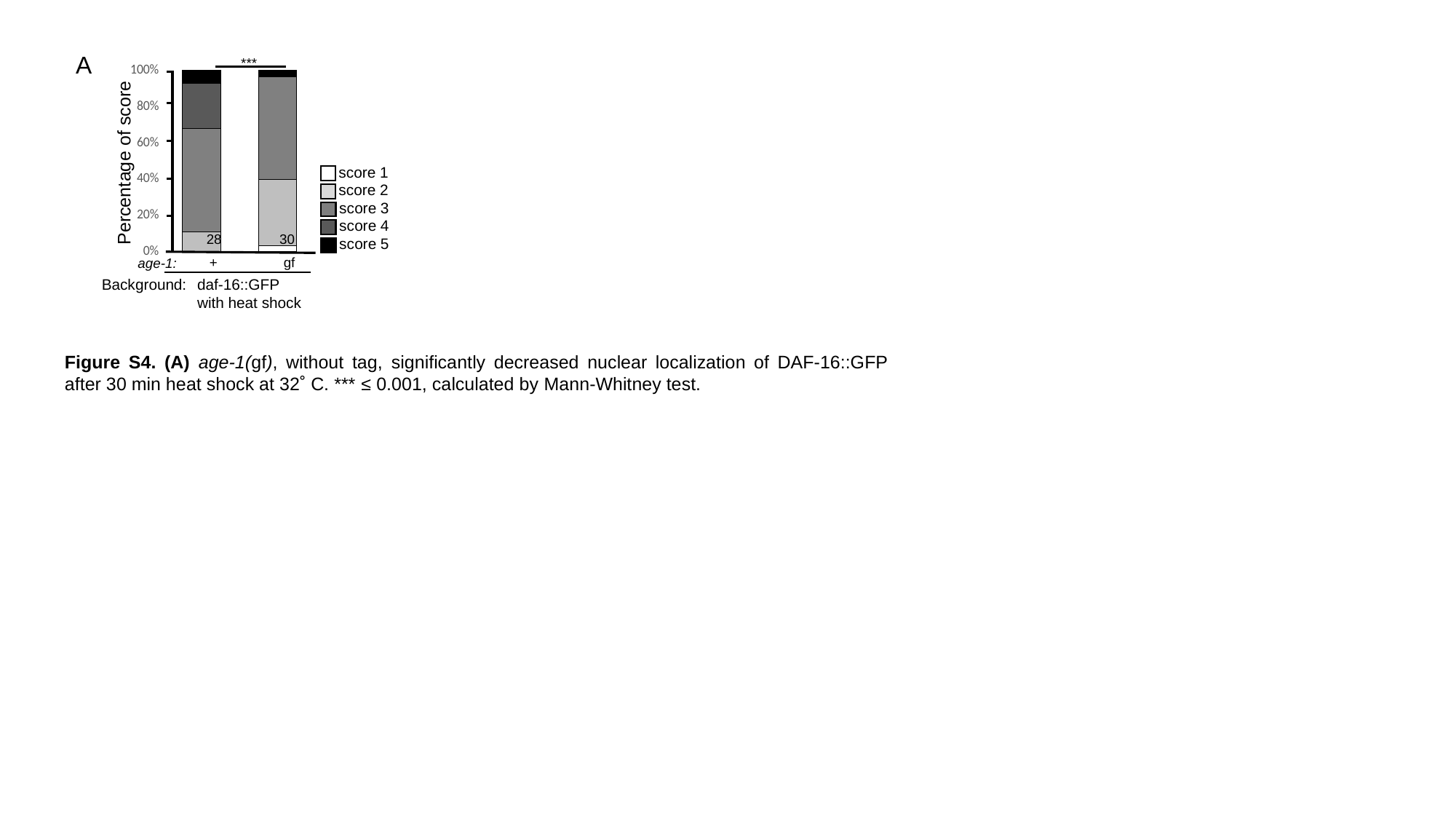

### Chart
| Category | score1 | score2 | score3 | score4 | score5 |
|---|---|---|---|---|---|
| daf-16 HS | 0.0 | 3.0 | 16.0 | 7.0 | 2.0 |
| age-1 gf;daf-16 HS | 1.0 | 11.0 | 17.0 | 0.0 | 1.0 |A
***
28
30
+
gf
daf-16::GFP
with heat shock
Percentage of score
score 1
score 2
score 3
score 4
score 5
age-1:
Background:
Figure S4. (A) age-1(gf), without tag, significantly decreased nuclear localization of DAF-16::GFP after 30 min heat shock at 32˚ C. *** ≤ 0.001, calculated by Mann-Whitney test.

## Slide 8
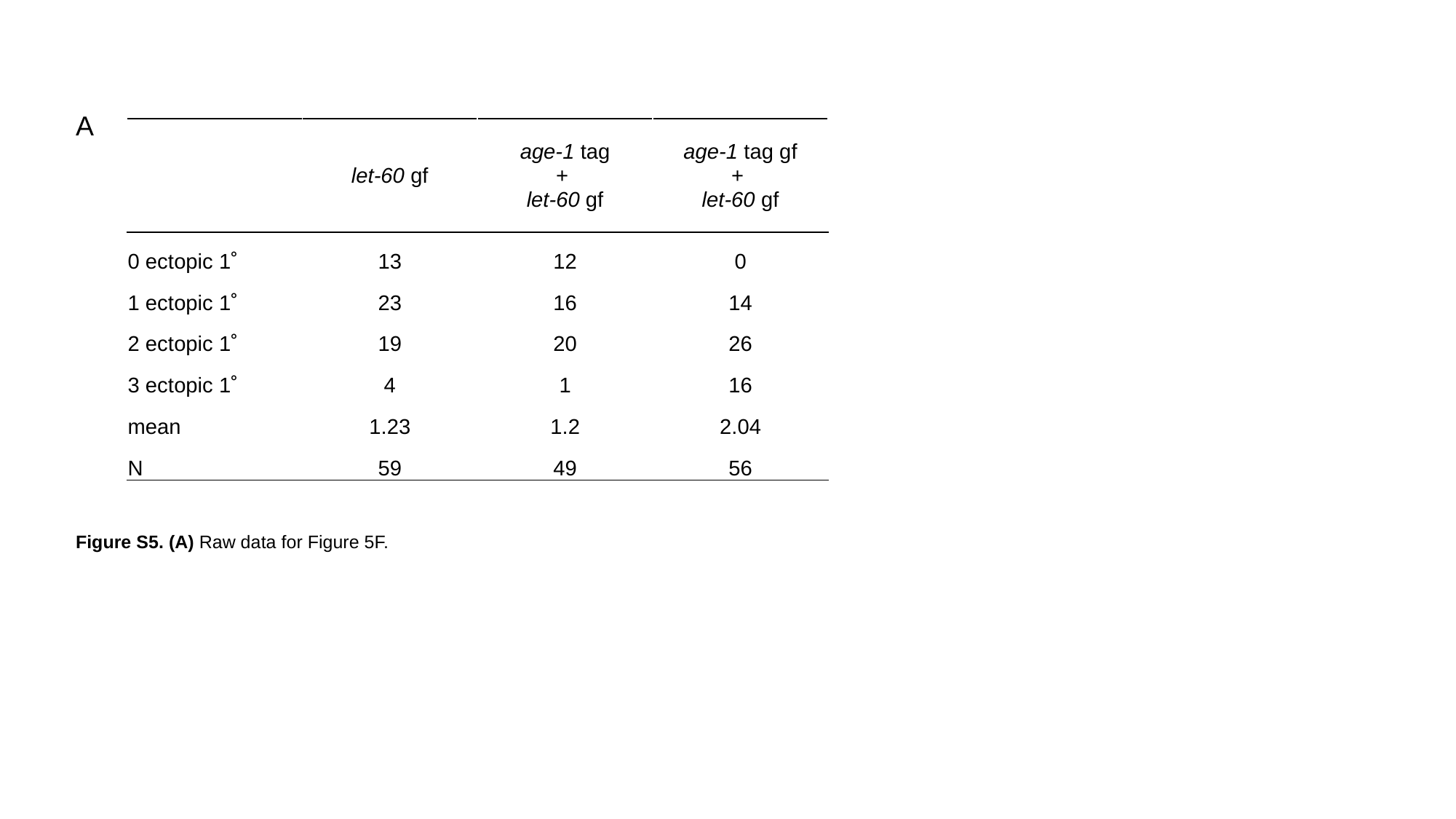

A
| | let-60 gf | age-1 tag + let-60 gf | age-1 tag gf + let-60 gf |
| --- | --- | --- | --- |
| 0 ectopic 1˚ | 13 | 12 | 0 |
| 1 ectopic 1˚ | 23 | 16 | 14 |
| 2 ectopic 1˚ | 19 | 20 | 26 |
| 3 ectopic 1˚ | 4 | 1 | 16 |
| mean | 1.23 | 1.2 | 2.04 |
| N | 59 | 49 | 56 |
Figure S5. (A) Raw data for Figure 5F.

## Slide 9
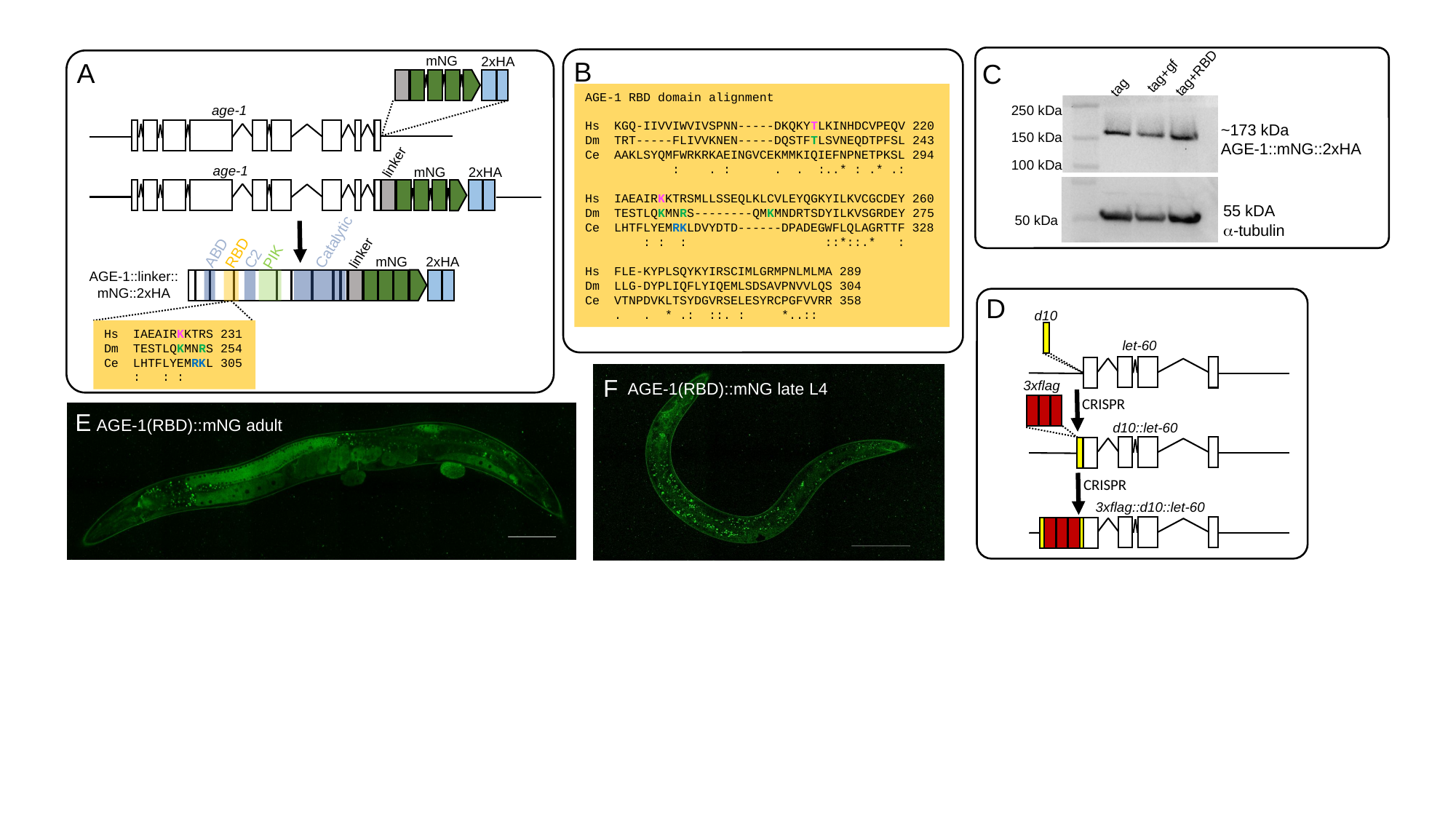

C
tag+RBD
tag+gf
tag
250 kDa
~173 kDa
AGE-1::mNG::2xHA
150 kDa
100 kDa
55 kDA
a-tubulin
50 kDa
mNG
2xHA
A
age-1
linker
age-1
2xHA
mNG
Catalytic
ABD
RBD
C2
linker
PIK
2xHA
mNG
AGE-1::linker:: mNG::2xHA
Hs IAEAIRKKTRS 231
Dm TESTLQKMNRS 254
Ce LHTFLYEMRKL 305
 : : :
B
AGE-1 RBD domain alignment
Hs KGQ-IIVVIWVIVSPNN-----DKQKYTLKINHDCVPEQV 220
Dm TRT-----FLIVVKNEN-----DQSTFTLSVNEQDTPFSL 243
Ce AAKLSYQMFWRKRKAEINGVCEKMMKIQIEFNPNETPKSL 294
 : . : . . :..* : .* .:
Hs IAEAIRKKTRSMLLSSEQLKLCVLEYQGKYILKVCGCDEY 260
Dm TESTLQKMNRS--------QMKMNDRTSDYILKVSGRDEY 275
Ce LHTFLYEMRKLDVYDTD------DPADEGWFLQLAGRTTF 328
 : : : ::*::.* :
Hs FLE-KYPLSQYKYIRSCIMLGRMPNLMLMA 289
Dm LLG-DYPLIQFLYIQEMLSDSAVPNVVLQS 304
Ce VTNPDVKLTSYDGVRSELESYRCPGFVVRR 358
 . . * .: ::. : *..::
D
d10
let-60
3xflag
CRISPR
d10::let-60
CRISPR
3xflag::d10::let-60
F
AGE-1(RBD)::mNG late L4
E
AGE-1(RBD)::mNG adult

## Slide 10
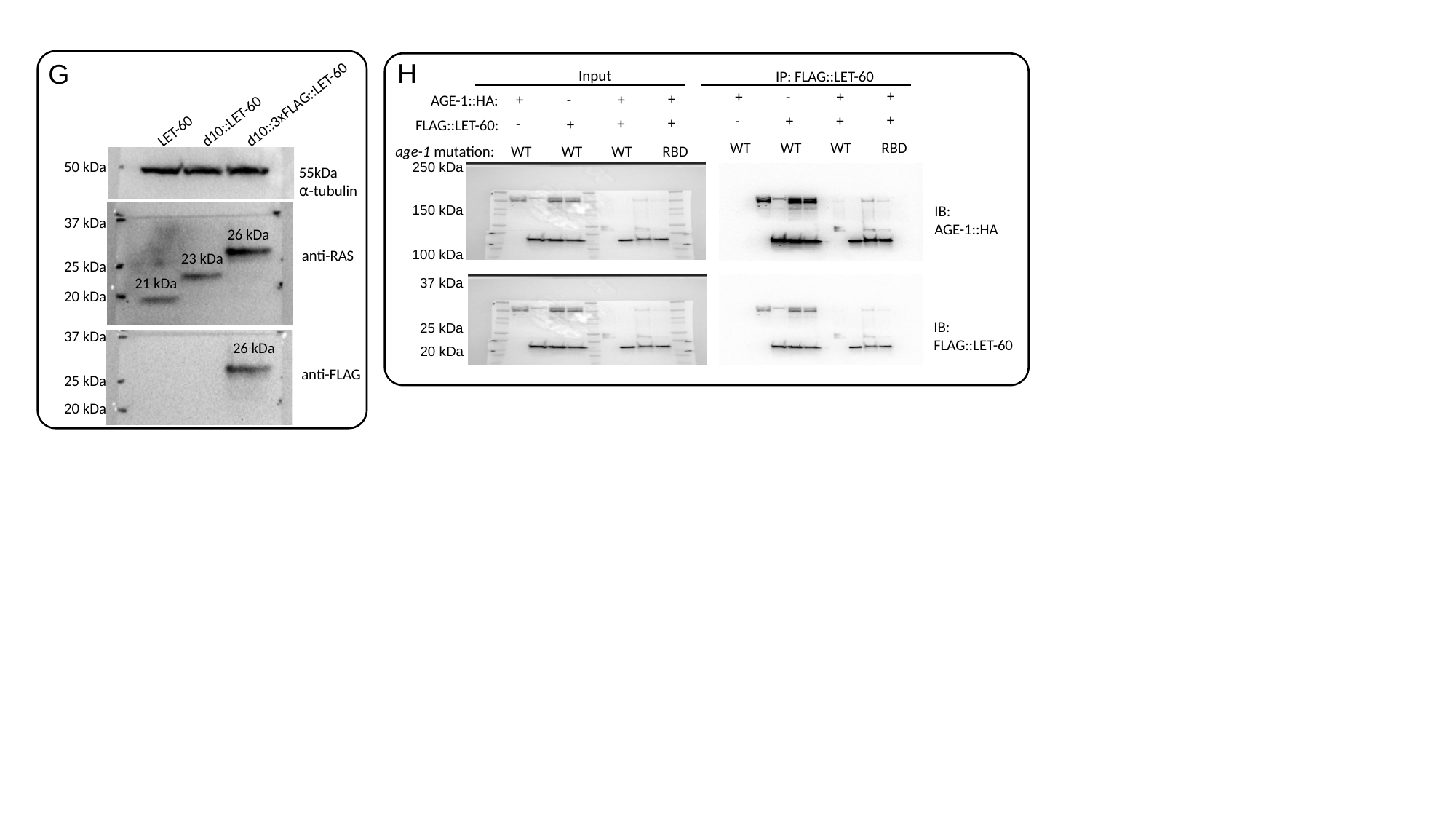

G
d10::LET-60
d10::3xFLAG::LET-60
LET-60
50 kDa
55kDa
⍺-tubulin
37 kDa
26 kDa
anti-RAS
23 kDa
21 kDa
25 kDa
20 kDa
37 kDa
26 kDa
anti-FLAG
25 kDa
20 kDa
H
Input
IP: FLAG::LET-60
+
+
-
+
+
+
-
+
AGE-1::HA:
+
-
+
+
+
-
+
+
FLAG::LET-60:
WT
WT
WT
RBD
age-1 mutation:
WT
WT
WT
RBD
250 kDa
150 kDa
IB:
AGE-1::HA
100 kDa
37 kDa
IB:
FLAG::LET-60
25 kDa
20 kDa

## Slide 11
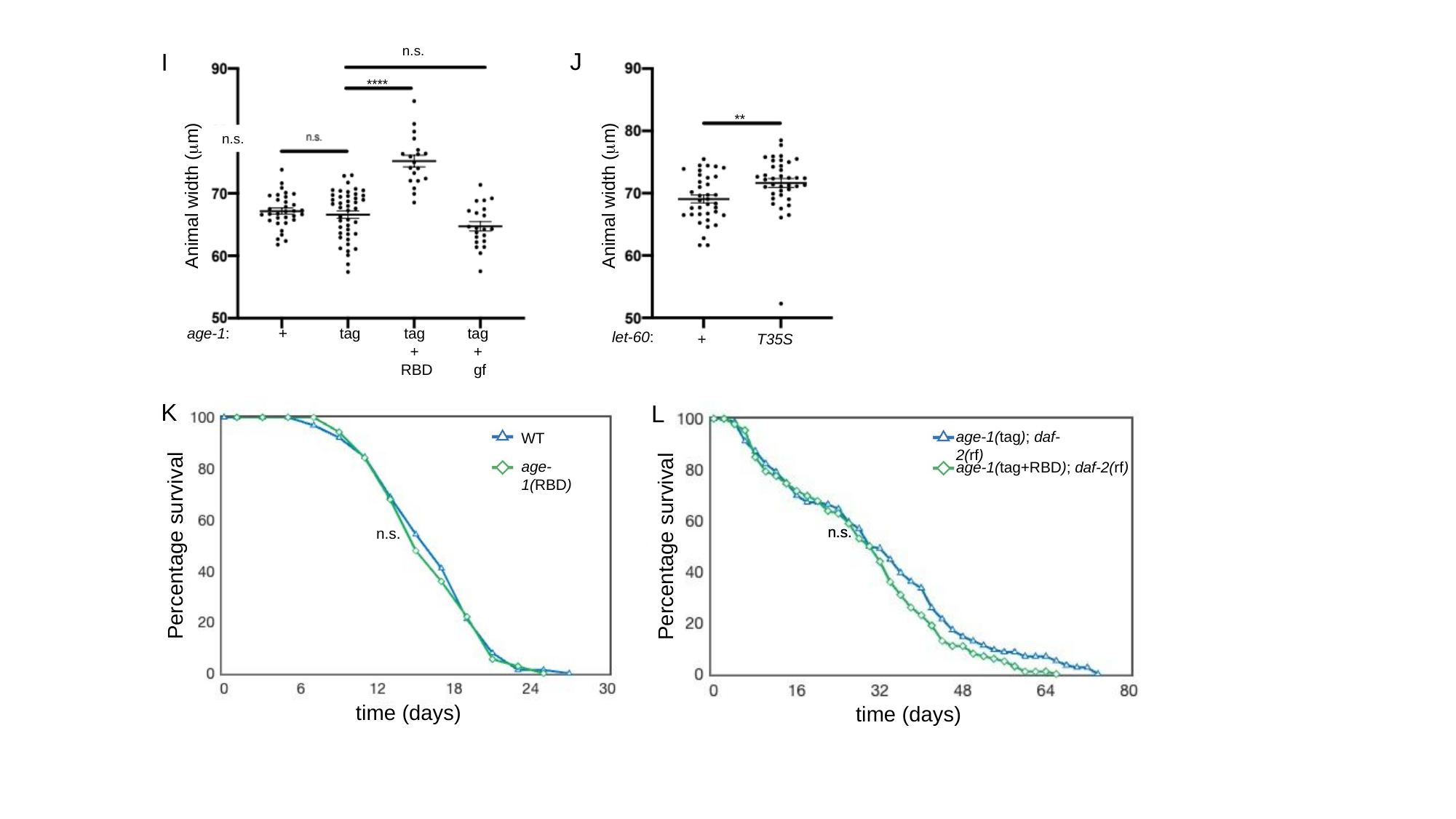

H
n.s.
J
I
Animal width (mm)
let-60:
+
T35S
**
****
n.s.
Animal width (mm)
age-1:
+
tag
tag
+
RBD
tag
+
gf
K
L
age-1(tag); daf-2(rf)
WT
age-1(RBD)
age-1(tag+RBD); daf-2(rf)
Percentage survival
Percentage survival
n.s.
n.s.
n.s.
time (days)
time (days)

## Slide 12
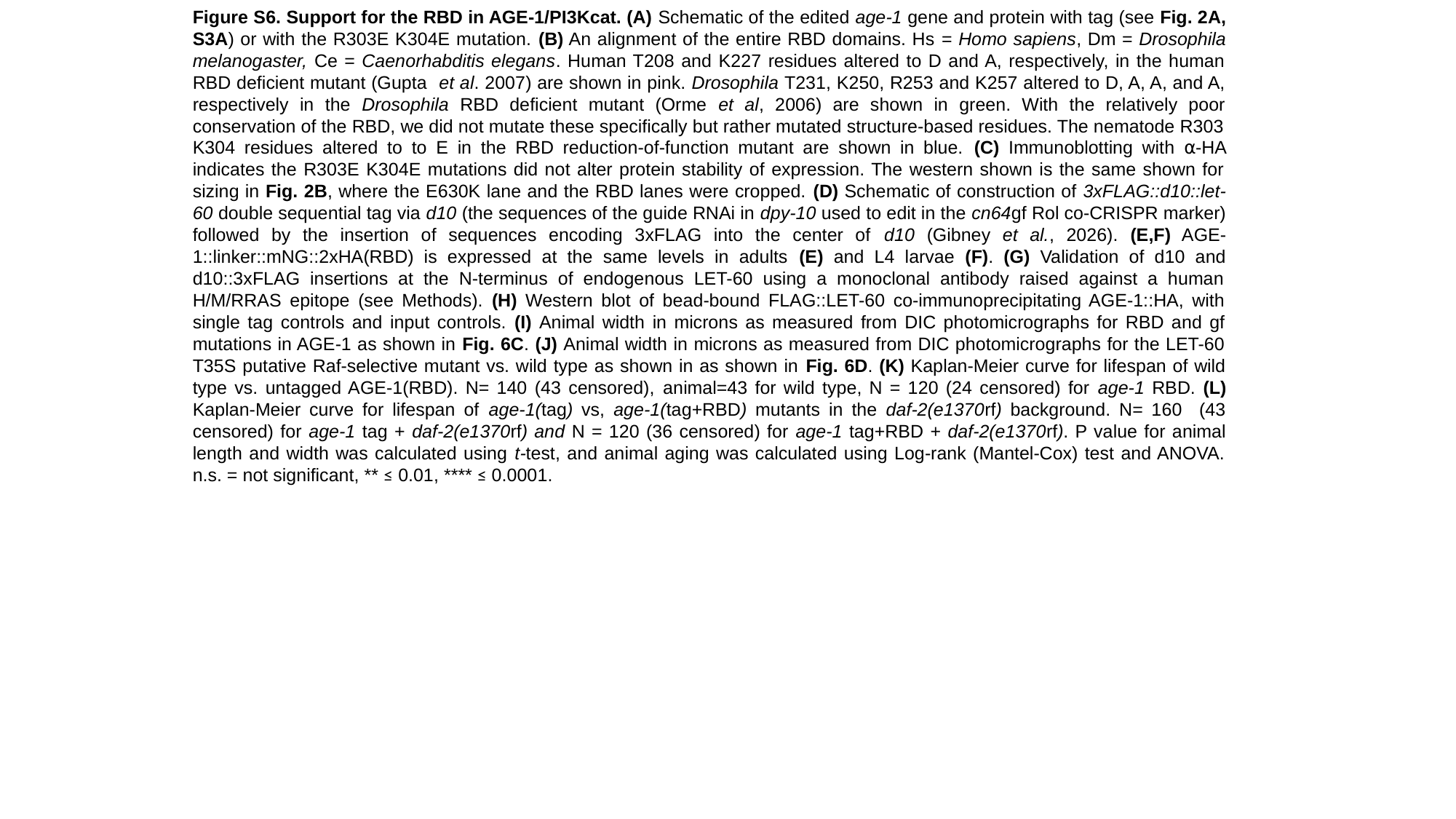

Figure S6. Support for the RBD in AGE-1/PI3Kcat. (A) Schematic of the edited age-1 gene and protein with tag (see Fig. 2A, S3A) or with the R303E K304E mutation. (B) An alignment of the entire RBD domains. Hs = Homo sapiens, Dm = Drosophila melanogaster, Ce = Caenorhabditis elegans. Human T208 and K227 residues altered to D and A, respectively, in the human RBD deficient mutant (Gupta et al. 2007) are shown in pink. Drosophila T231, K250, R253 and K257 altered to D, A, A, and A, respectively in the Drosophila RBD deficient mutant (Orme et al, 2006) are shown in green. With the relatively poor conservation of the RBD, we did not mutate these specifically but rather mutated structure-based residues. The nematode R303 K304 residues altered to to E in the RBD reduction-of-function mutant are shown in blue. (C) Immunoblotting with ⍺-HA indicates the R303E K304E mutations did not alter protein stability of expression. The western shown is the same shown for sizing in Fig. 2B, where the E630K lane and the RBD lanes were cropped. (D) Schematic of construction of 3xFLAG::d10::let-60 double sequential tag via d10 (the sequences of the guide RNAi in dpy-10 used to edit in the cn64gf Rol co-CRISPR marker) followed by the insertion of sequences encoding 3xFLAG into the center of d10 (Gibney et al., 2026). (E,F) AGE-1::linker::mNG::2xHA(RBD) is expressed at the same levels in adults (E) and L4 larvae (F). (G) Validation of d10 and d10::3xFLAG insertions at the N-terminus of endogenous LET-60 using a monoclonal antibody raised against a human H/M/RRAS epitope (see Methods). (H) Western blot of bead-bound FLAG::LET-60 co-immunoprecipitating AGE-1::HA, with single tag controls and input controls. (I) Animal width in microns as measured from DIC photomicrographs for RBD and gf mutations in AGE-1 as shown in Fig. 6C. (J) Animal width in microns as measured from DIC photomicrographs for the LET-60 T35S putative Raf-selective mutant vs. wild type as shown in as shown in Fig. 6D. (K) Kaplan-Meier curve for lifespan of wild type vs. untagged AGE-1(RBD). N= 140 (43 censored), animal=43 for wild type, N = 120 (24 censored) for age-1 RBD. (L) Kaplan-Meier curve for lifespan of age-1(tag) vs, age-1(tag+RBD) mutants in the daf-2(e1370rf) background. N= 160 (43 censored) for age-1 tag + daf-2(e1370rf) and N = 120 (36 censored) for age-1 tag+RBD + daf-2(e1370rf). P value for animal length and width was calculated using t-test, and animal aging was calculated using Log-rank (Mantel-Cox) test and ANOVA. n.s. = not significant, ** ≤ 0.01, **** ≤ 0.0001.
