# Supplementary material for: Aging, Dauer, and Stature Phenotypes Are Conferred by Structure‐Directed Missense Mutations in the Endogenous AGE‐1/Phosphatidylinositol 3‐Kinase Catalytic Subunit
Source: Aging Cell. 2026 Jun 18;25(6):e70571. doi: 10.1111/acel.70571 (PMC13277760; doi:10.1111/acel.70571)
Supplement: Supplementary file 2 — Table S1: Strain list. Table S2: Primer list. Table S3: CRISPR reagents. [file ACEL-25-e70571-s002.docx]

**Table S1. Strain list**

| **Strain** | **Genotype** | **Impact** |
| --- | --- | --- |
| DV4018 | *age-1(re353*tag*[age-1::mNG::2xHA])* II | FP+epitope |
| DV4070 | *age-1(*re353re377tag+RBD*[age-1::mNG::2xHA(*R303E,K304E*)])* II | RBD |
| DV4138 | *age-1(re353re392*gf*[*E630K*])* II | activated |
| DV4019 | *age-1(re353*tag*)* II; *daf-2(m577*rf*)* III |  |
| GR1318 | *pdk-1*(*mg142*gf*)* X | activated |
| DV4179 | *age-1*(*re353re392*gf) II; *daf-2*(*e1370*rf) III |  |
| DV4180 | *age-1*(*re353*tag) II; *daf-2*(*e1370*rf) III |  |
| DV4181 | *age-1*(*re353re377*tag+RBD) II; *daf-2*(*e1370rf*) III |  |
| DV4019 | *age-1(re353*tag*)* II; *daf-2*(*m577*rf) III |  |
| DV4075 | *age-1(re353re377*tag+RBD*)* II; *daf-2(m577*rf*)* III |  |
| DV4139 | *age-1(*re353re392tag+gf*)* II; *daf-2*(*m577*rf) III |  |
| DV4094 | *let-60*(*re378[*T35S*]*) IV | effector |
| DV4112 | *daf-2*(*m577*rf) III; *let-60*(r*e378[*T35S*]*) IV |  |
| DV4300 | *age-1(re353*tag*)* II; *let-60(n1046*gf*)* IV |  |
| DV4301 | *age-1(re353re392*tag+gf) II; *let-60(n1046*gf*)* IV |  |
| DV4302 | *age-1(re353re377*tag*+*RBD*)* II; *let-60(n1046*gf*)* IV |  |
| DV4492 | *let-60(re302re498[d10::3xFLAG::let-60])* IV | epitope |
| DV4493 | *age-1(re353[*tag*])* II*; let-60(re302re498[d10::3xFLAG::let-60])* IV |  |
| DV4494 | *age-1(re353re377[*tag+RBD*])* II*; let-60(re302re498[d10::3xFLAG::let-60])* IV |  |
| DV4445 | *age-1(re489*gf*[*E630K*])* II |  |
| DV4483 | *age-1(re489*gf*[*E630K*])* II*; zIs356[daf-16p>daf-16a/b::GFP+rol-6(su1006*d*)]* IV |  |
| DV4484 | *age-1(re489*gf*[*E630K*])* II*; daf-2(m577*rf*)* III*; zIs356 [daf-16p>daf-16a/b::GFP+rol-6(su1006*d*)]* IV |  |
| DV4485 | *daf-2(m577)* III*; zIs356 [daf-16p>daf-16a/b::GFP+rol-6(su1006*d*)]* IV |  |
| DV4446 | *age-1(re376re490*gf+RBD*[age-1(R303E K304E E630K)])* II |  |
| DV4457 | *age-1(re489*gf*[*E630K*])* II*; daf-2(m577*rf*)* III |  |
| DV4458 | *age-1(re376re490*gf+RBD*[age-1(R303E K304E E630K)])* II*; daf-2(m577*rf*)* III |  |

**Table 2. Primer list**

| **Primer name** | **Sequence (5’-3’)** | **Used for** |
| --- | --- | --- |
| YW_56 | cgtatttctagagaaaaaaacagtctttcgtctggg | *age-1(re353*tag*)* genotyping |
| YW_57 | TCACTGATGTTATGGGAATGGACGAGC | *age-1(re353*tag*)* genotyping |
| YW_58 | aataaacacagggagagaggagagagagg | *age-1(re353*tag*)* genotyping |
| YW_96 | GTGCTTCTCTCTGGGCTC | *age-1(re353re377*RBD*)* genotyping |
| YW_97 | GGTATCGTATACATCCAATTTTCGCATT | *age-1(re353re377*RBD*)* genotyping |
| YW_98 | CTACGAGATGGAGGAGCTAGAC | *age-1(re353re377*RBD*)* genotyping |
| YW_99 | AGAACAACGAATCCAGGGC | *age-1*(*re353re377*RBD) genotyping |
| YW_108 | CTTTTGATCTATACATGAAGGATATGCCACC | *age-1(*r*e353re392*gf*)* genotyping |
| YW_109 | ATCCAGACATGACGTTGTTCATCTTC | *age-1(re353re392*gf*)* & *age-1(re489*gf*)* genotyping |
| YW_110 | CATAGTGCTGGAAAAGGACGAACA | *age-1(re353re392*gf*)* & *age-1(re489*gf*)* genotyping |
| YW_111 | CAACGCCTGTATGAGAGGCAATATG | *age-1(re353re392*gf*)* & *age-1(re489*gf*)* genotyping |
| DJR_879 | gtatgccgataggcagagctgc | *let-60(re378[*T35S*])* genotyping  *Snip-SNP with added PvuI* |
| DJR_880 | gtgtttcaaaaaacttccaaacttctgactcatcg | *let-60(re378[*T35S*])* genotyping  *Snip-SNP with added PvuI* |
| YW_302 | tctttcctggtagttactctcctttatcg | *let-60(re302re498[d10::3xFLAG::let-60])* genotyping |
| YW_303 | GCTTTCTGTAGCTGTCCTCTATGG | *let-60(re302re498[d10::3xFLAG::let-60])* genotyping |
| YW_304 | CGACAAGCGTGATTACAAGGATG | *let-60(re302re498[d10::3xFLAG::let-60])* genotyping |

**Table 3. CRISPR reagents**

| **Name** | **Sequence (5’-3’)** | **Used for** |
| --- | --- | --- |
| TD_479 | UUCAGUAGUGUUUGACUGCGGUUUUAGAGCUAUGCUGUUUUG | crRNA for *age-1* tag |
| TD_478 | TCATGGTCTACCAAAACGAATTGGCTCTTCCACGCAGTCAAACACTACGGGGCATCGGGAGCATCGGCGGGAGGTTGTTCCGGGGCGGGGGGATCAGGTGGCTCCGCAGGGGCGTCCGGATGTGGCGGAGCGTGTTCGATGGTCAGCAAAGGCGAGGAAGACAACATGGCTTCTCTTCCAGCAACTCACGAGCTTCACATTTTCGGATCCATCAACGGAGTCGATTTCGATATGGTTGGACAGGGAACTGGAAATCCAAATGACGGTTACGAAGAGCTCAACTTAAAATCGACGAAGgtaagtttaaacatatatatactaactaaccctgattatttaaattttcagGGAGACCTTCAATTCTCACCATGGATTTTAGTGCCCCACATTGGATACGGATTTCATCAATATCTTCCATATCCAGATGGAATGAGTCCTTTTCAAGCCGCTATGGTCGATGGATCCGGATACCAAGTTCATCGAACAATGCAATTCGAAGgtaagtttaaacagttcggtactaactaaccatacatatttaaattttcagACGGAGCTTCCCTTACTGTAAACTATCGTTATACCTATGAGGGGTCCCACATCAAGGGCGAGGCTCAAGTCAAGGGAACTGGATTCCCAGCCGATGGACCAGTTATGACCAACAGTCTCACCGCCGCCGATTGGTGCCGCTCCAAGAAGACCTATCCAAATGACAAGACCATCATTTCAACATTCAAATGGTCTTACACAACTGGAAACGGAAAAAGgtaagtttaaacatgattttactaactaactaatctgatttaaattttcagATACCGATCGACAGCTCGTACAACGTACACGTTCGCCAAACCAATGGCTGCCAACTATTTGAAAAACCAGCCAATGTACGTGTTCCGTAAGACCGAGCTTAAGCACTCTAAAACTGAATTAAACTTCAAGGAGTGGCAAAAAGCTTTCACTGATGTTATGGGAATGGACGAGCTGTACGGAGCCGGATCTTATCCATACGATGTCCCAGATTACGCTTACCCATATGACGTTCCAGACTATGCCTGAaacctctgttatctaataatataacacattcc | gBlock for *age-1* tag repair oligo |
| YW_87 | UCUCUACGAAAUGCGAAAAUGUUUUAGAGCUAUGCUGUUUUG | crRNA for *age-1(re353re377*rf*)* |
| YW_86 | ACTCCGAAATCTCTGCTTCACACGTTTCTCTACGAGATGGAGGAGCTAGACGTATACGATACCGATGATCCTGCAGATGAAGGATG | Repair template for *age-1(re353re377*rf*)* |
| YW_107 | UGAAUCUGGAAUUGUAUUAGGUUUUAGAGCUAUGCUGUUUUG | crRNA for *age-1(re353re392*gf*)* |
| YW_106 | AAGCTTCAGATGCTTGTCAAGAAGCATGAATCTGGCATCGTGCTGGAAAAGGACGAACAACGTCATGTCTGGATGTGGAGGAGATACAT | Repair template for *age-1(re353re392*gf*)* |
| DJR_884 | AGAAUACGACCCGACCAUAGGUUUUAGAGCUAUGCUGUUUUG | crRNA for *let-60(re378[*T35S*])* |
| DJR_885 | TTCAACTCATCCAGAATCACTTTGTCGAAGAATACGACCCGTCGATCGAGGACAGCTACAGAAAGCAGgtgagaaatcattgggaacatc | Repair template for *let-60(re378[*T35S*])* |
| YW_301 | gggtaATGACGGAGTACAAGGGAAACCGTACCGCTGATTATAAAGACGATGACGATAAGCGTGACTACAAGGACGACGACGACAAGCGTGATTACAAGGATGACGATGACAAGAGAC^TGGTGCCTATGGTAGCGGAGCTTCAATGACGGAG | Repair template for *let-60(re302re498[d10::3xFLAG::let-60])* |
